# Supplementary material for: Sequence-encoded and composition-dependent protein-RNA interactions control multiphasic condensate morphologies
Source: Nat Commun. 2021 Feb 8;12:872. doi: 10.1038/s41467-021-21089-4 (PMC7870978; doi:10.1038/s41467-021-21089-4)
Supplement: Supplementary file 1 — Supplementary Information [file 41467_2021_21089_MOESM1_ESM.pdf]

Supplementary Information for

Sequence-encoded and Composition-dependent Protein-RNA Interactions Control Multiphasic Condensate Morphologies

Taranpreet Kaur[1], Muralikrishna Raju[2], Ibraheem Alshareedah[1], Richoo B. Davis[1], Davit A. Potoyan[2]*, Priya R. Banerjee[1]*

[1]Department of Physics, University at Buffalo, Buffalo NY 14260, USA

[2]Department of Chemistry, Iowa State University, Ames IA 50011, USA

These authors contributed equally: Muralikrishna Raju, Ibraheem Alshareedah

*Corresponding Authors:

Priya R. Banerjee: prbanerj@buffalo.edu

Davit A. Potoyan: potoyan@iastate.edu

Supplementary Tables

Protein/ polypeptide	N-terminal Tag	Purpose of the N-terminal Tag/ site-directed mutagenesis	Extinction Coefficient ($M^{-1}\cdot cm^{-1}$)
FUS	His6-MBP-N10	Purification	138230
PLP of FUS (FUS^{PLP})	His6-MBP-N10	Purification	103600
FUS S86C	His6-MBP-N10	Purification/ Cys-maleimide conjugation used for site-specific protein labeling	138230
FUS^{PLP} S86C	His6-MBP-N10	Purification/ Cys-maleimide conjugation used for site-specific protein labeling	103600
EWS^{PLP} A2C	His6-MBP-N10	Purification/ Cys-maleimide conjugation used for site-specific protein labeling	122970
RNA Pol II^{CTD} 2C	His6-MBP-N10	Purification/ Cys-maleimide conjugation used for site-specific protein labeling	112540
$BRG1^{LCD}$ 2C	His6-MBP-N10	Purification/ Cys-maleimide conjugation used for site-specific protein labeling	80790

Table S1. List of the proteins used in the study. Also, see Table S2 for their amino acid sequences. The molar extinction coefficients were calculated using ProtParam[1].

**Protein/Peptide**	**Sequence**
FUS^{PLP} (PLP)	MASNDYTQQATQSYGAYPTQPGQGYSQQSSQPYGQQSYSGYSQSTDTSGYG<br>QSSYSSYGQSQNSYGTQSTPQGYGSTGGYGSSQSSQSSYGQQSSYPGYGQQ<br>PAPSSTSGSYGSSSQSSSYGQPQSGSYSQQPSYGGQQQSYGQQQSYNPPQG<br>YGQQNQYNSSSGGGGGGGGG
EWS^{PLP}	MASTDYSTYSQAAAQQGYSAYTAQPTQGYAQTTQAYGQQSYGTYGQPTDVSY<br>TQAQTTATYGQTAYATSYGQPPTGYTTPTAPQAYSQPVQGYGTGAYDTTTATV<br>TTTQASYAAQSAYGTQPAYPAYGQQPAATAPTRPQDGNKPTETSQPQSSTGGY<br>NQPSLGYGQSNYSYPQVPGSYPMQPVTAPPSYPPTSYSSTQPTSYDQSSYSQ<br>QNTYGQPSSYGQQSSYGQQSSYGQQPPTSYPPQTGSYSQAPSQYSQQSSSY<br>GQQS
RNA Pol II CTD	MYSPTSPAYEPRSPGGYTPQSPSYSPTSPSYSPTSPSYSPTSPNYSPTSPSYSP<br>TSPSYSPTSPSYSPTSPSYSPTSPSYSPTSPSYSPTSPSYSPTSPSYSPTSPSYS<br>PTSPSYSPTSPSYSPTSPSYSPTSPSYSPTSPSYSPTSPSYSPTSPNYSPTSPNY<br>TPTSPSYSPTSPSYSPTSPNYTPTSPNYSPTSPSYSPTSPSYSPTSPS
FUS^{FL}	MASNDYTQQATQSYGAYPTQPGQGYSQQSSQPYGQQSYSGYSQSTDTSGYG<br>QSSYSSYGQSQNSYGTQSTPQGYGSTGGYGSSQSSQSSYGQQSSYPGYGQQ<br>PAPSSTSGSYGSSSQSSSYGQPQSGSYSQQPSYGGQQQSYGQQQSYNPPQG<br>YGQQNQYNSSSGGGGGGGGGGNYGQDQSSMSSGGGSGGGYGNQDQSGGG<br>GSGGYGQQDRGGRGRGGSGGGGGGGGGGGYNRSSGGYEPRGRGGGRGGR<br>GGMGGSDRGGFNKFGGPRDQGSRHDSEQDNSDNNTIFVQGLGENVTIESVAD<br>YFKQIGIIKTNKKTGQPMINLYTDRETGKLKGEATVSFDDPPSAKAAIDWFDGKEF<br>SGNPIKVSFATRRADFNRGGGNGRGGRGRGGPMGRGGYGGGGSGGGGRGG<br>FPSGGGGGGGQQRAGDWKCPNPTCENMNFSWRNECNQCKAPKPDGPGGGP<br>GGSHMGGNYGDDRRGGRGGYDRGGYRGRGGDRGGFRGGRGGGDRGGFGP<br>GKMDSRGEHRQDR RERPY
BRG1^{LCD}	MSTPDPPLGGTPRPGPSPGPGPSPGAMLGPSPGPSPGSAHSMMGPSPGPPSA<br>GHPIPTQGPGGYPQDNMHQMHKPMESMHEKGMSDDPRYNQMKGMGMRSGG<br>HAGMGPPPSPMDQHSQGYPSPLGGSEHASSPVPASGPSSGPQMSSGPGGAP<br>LDGADPQALGQQNRGPTPFNQNQLHQLRAQIMAYKMLARGQPLPDHLQMAVQ<br>GKRPMPGMQQQMPTLPPPSVSATGPGPGPGPGPGPGPGPAPPNYSRPHGMG<br>GPNMPPPGPSGVPPGMPGQPPGGPPKPWPEGPMANAAAPTSTPQKLIPPQPT<br>GRPSPAPPAVPPAASPVMPPQTQSPGQPAQPA
[RGRGG]$_5$	RGRGG RGRGG RGRGG RGRGG RGRGGC
[KGKGG]$_5$	KGKGG KGKGG KGKGG KGKGG KGKGGC
[KGYGG]$_5$	KGYGG KGYGG KGYGG KGYGG KGYGGC
FUS^{RGG3}	RRGGRGGYDRGGYRGRGGDRGGFRGGRGGGDRGC
**Peptide/RNA**	**Sequences utilized in simulation**
RRP (FUS^{RGG3})	RRGGRGGYDRGGYRGRGGDRGGFRGGRGGGDRGC
RNA	$[U]_{100}$
PLP	MASNDYTQQATQSYGAYPTQPGQGYSQQSSQPYGQQSYSGYSQSTDTSGYG<br>QSSYSSYGQSQNSYGTQSTPQGYGSTGGYGSSQSSQSSYGQQSSYPGYGQQ<br>PAPSSTSGSYGSSSQSSSYGQPQSGSYSQQPSYGGQQQSYGQQQSYNPPQG<br>YGQQNQYNSSSGGGGGGGGG
FUS polypeptide	MASNDYTQQATQSYGAYPTQPGQGYSQQSSQPYGQQSYSGYSQSTDTSGYG<br>QSSYSSYGQSQNSYGTQSTPQGYGSTGGYGSSQSSQSSYGQQSSYPGYGQQ<br>PAPSSTSGSYGSSSQSSSYGQPQSGSYSQQPSYGGQQQSYGQQQSYNPPQG<br>YGQQNQYNSSSGGGGGGGGGRRGGRGGYDRGGYRGRGGDRGGFRGGRGG<br>GDRGC

Table S2. Amino acid sequences of the proteins and peptides used in the study. Highlighted residues are Tyrosine (red) and Arginine (blue) residues.

Supplementary Figures

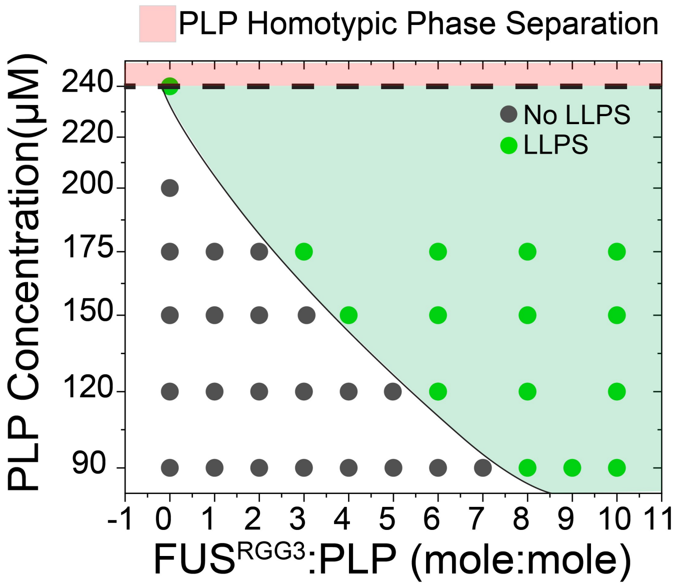

Figure S1. FUS^{RGG3}-PLP isothermal state diagram. A state diagram for PLP-FUS^{RGG3} mixtures, showing that FUS^{RGG3} facilitates PLP (FUS^{PLP}) phase separation. The shaded green region shows the co-phase separation regime for PLP-FUS^{RGG3} mixtures while the shaded pink region denotes PLP homotypic phase separation regime (saturation concentration ~240 μM). Both shaded regions are drawn as a guide to the eye. The sample buffer contains 25 mM Tris-HCl (pH 7.5), 150 mM NaCl and 20 mM DTT.

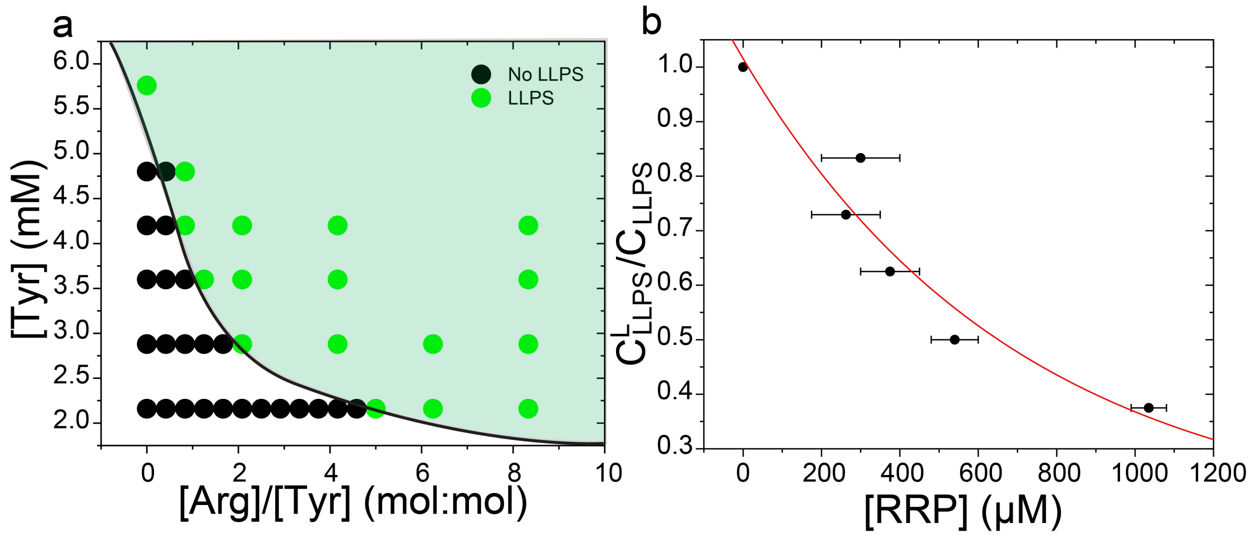

Figure S2. [RGRGG]$_5$-PLP isothermal state diagram. (a) A state diagram for [RGRGG]$_5$-PLP mixtures. This is identical data to Figure 1a, main-text, but plotted against tyrosine concentrations in PLP and arginine-to-tyrosine ratio. The shaded green region represents the phase-separation regime and is drawn as a guide to the eye. **(b)** A plot of the ratio of LLPS concentration threshold for PLP with ligand (C^L_{LLPS}) and without ligand (C_{LLPS}) as a function of [RRP] concentration (ligand = RRP). The data points are estimated from the state diagram analysis as shown in Figure 1a, main-text. At a fixed concentration of PLP (any horizontal line across Figure 1a main-text), the ligand concentration required for LLPS was estimated as the mid-point between the LLPS (green) and No LLPS (black) transition points. Here, the error bars are equal to half of the range between the two points that define a transition between mixed regime (no LLPS) and phase separation regime (LLPS). The black points represent the data, while the red line is drawn as a guide to the eye. RRP = [RGRGG]$_5$.

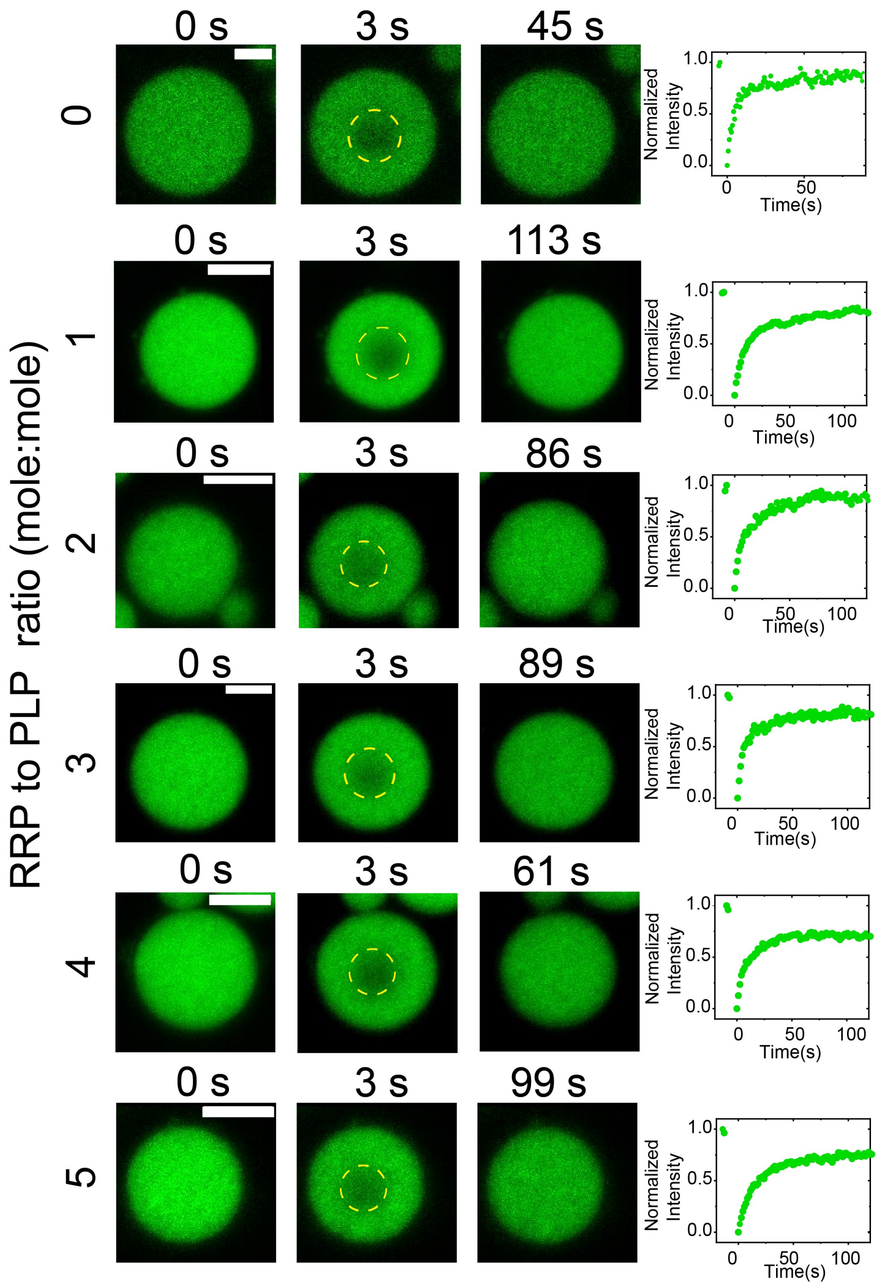

Figure S3. Representative experimental data for FRAP experiments on PLP-RRP condensates. Time-lapse FRAP images (left) and the corresponding intensity time traces (right) for PLP-RRP condensates prepared at a fixed FUS^{PLP} concentration of 280 μM and variable [RGRGG]$_5$-to-FUS^{PLP} ratios. The sample buffer contains 25 mM Tris-HCl (pH 7.5), 150 mM NaCl and 20 mM DTT. The yellow dashed circle indicates the predetermined bleaching region. Scale bars are 5 μm. Bleaching occurs at t=3s. The microscopy images are representative of at least three FRAP events from different spots in the same sample. The FRAP experiment was performed utilizing ~1% (labeled: unlabeled ratio) Alexa488-labeled PLP.

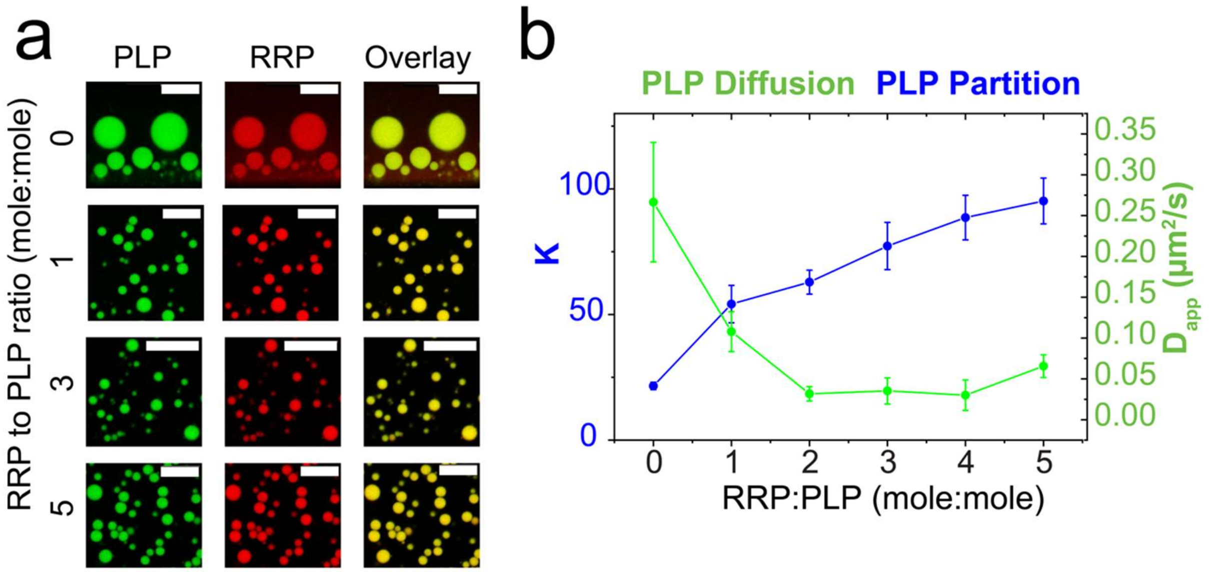

Figure S4. FUS^{PLP} partition and apparent diffusion are altered with increasing RRP concentration. (a) Multicolor confocal fluorescence microscopy images for PLP-RRP condensates at a variable RRP-to-PLP mixing ratio. We note that the difference in size between PLP droplets and PLP-RRP droplets may be a consequence of RRP-PLP condensates having excess charge due to the presence of Arg residues. For all samples, PLP concentration is fixed at 280 μM and RRP ($[RGRGG]_5$) concentration was varied. Scale bars represent 20 μm. ~500 nM Alexa488-labeled PLP and ~500 nM Alexa594-labeled $[RGRGG]_5$ were used for visualization. The reported images are representative of several replicates imaged from different spots in the same sample. **(b)** A plot showing PLP partition coefficient (K*;* n = 60 droplets per sample) and apparent diffusion coefficient (D_{app}; n = 3 droplets per sample) as a function of RRP-to-PLP mixing ratio. Error bars represent $\pm$ 1 s.d and the central filled circle represents the mean value. (see the statistical analysis section and Fig. 1b&c*,* main-text). Source data are provided as a Source Data file. The sample buffer contains 25 mM Tris-HCl (pH 7.5), 150 mM NaCl and 20 mM DTT.

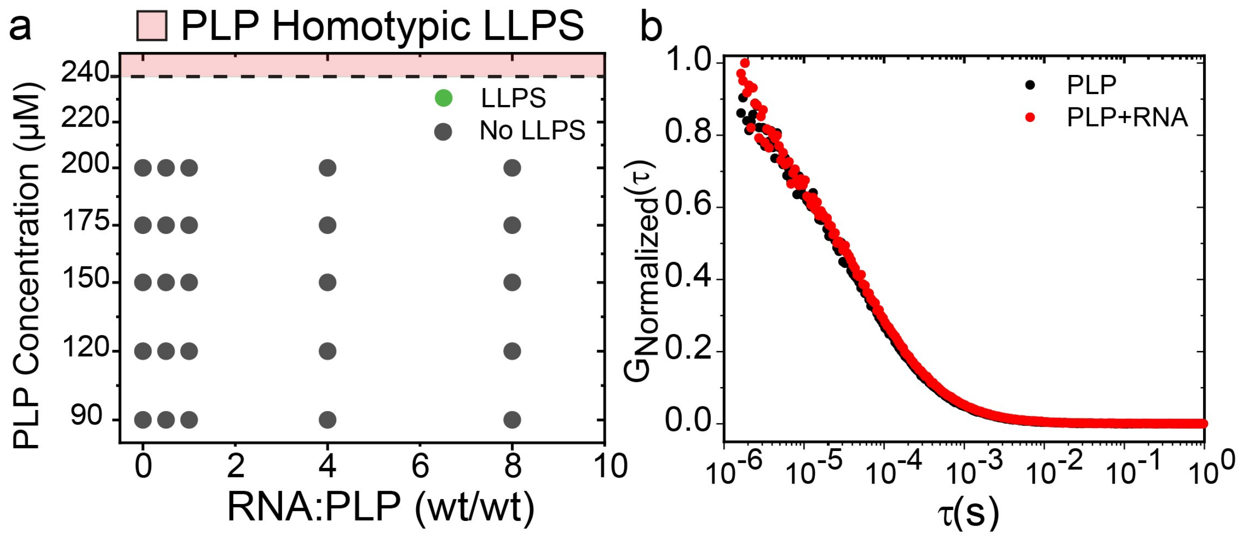

Figure S5. State diagram analysis and Fluorescence Correlation Spectroscopy (FCS) for PLP-RNA mixture. (a) State diagram of PLP-RNA mixtures, showing that poly(rU) RNA does not affect PLP phase-separation. Shaded pink region denotes PLP homotypic phase separation regime (PLP saturation concentration: C_{sat} ~ 240 μM). **(b)** The normalized autocorrelation curve for FUS^{PLP} in the presence (red) and absence of RNA (black). The time scale at which the autocorrelation reaches zero is proportional to the diffusion time of the labeled molecules. PLP shows identical auto-correlation time-scale both in the presence and absence of RNA, indicating that PLP is not forming a complex with RNA (which would slow the diffusion and therefore would change the autocorrelation timescale). The sample contained [Alexa488-labeled FUS^{PLP}]= 50 nM (0.88 ng/ml) with 0.0 ng/ml RNA poly(rU) (black) and 7.1 ng/ml RNA poly(rU) (red).The sample buffer contains 25 mM Tris-HCl (pH 7.5), 150 mM NaCl.

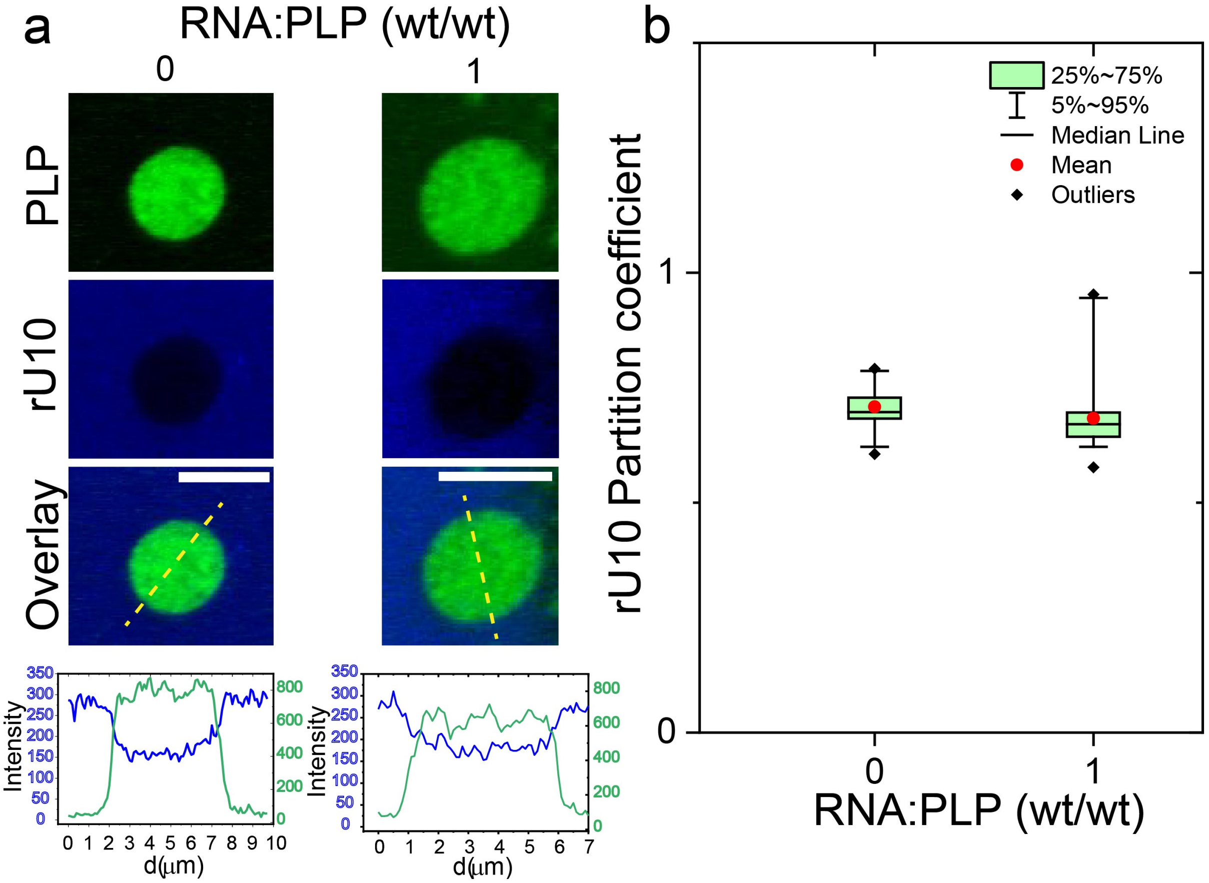

Figure S6. An RNA oligomer, rU10 remains excluded from PLP condensates. Multicolor confocal fluorescence microscopy images and intensity profiles (across yellow dashed lines) **(a)** and partition coefficients box plot **(b)** showing that FAM-labeled rU10 is excluded out of PLP droplets at the tested poly(rU)-to-PLP ratio. PLP condensates were prepared at PLP = 280 μM (with ~ 1% Cy-5-labeled PLP) and varying poly(rU)-to-PLP ratio. The number of droplets (n) analyzed across different samples for partition is n = 35. Source data are provided as a Source Data file.The microscopy images in (a) are representative of two independent sample replicates. Scale bars represent 5 μm. The sample buffer contains 25 mM Tris-HCl (pH 7.5) and 150 mM NaCl. PLP = FUS^{PLP}.

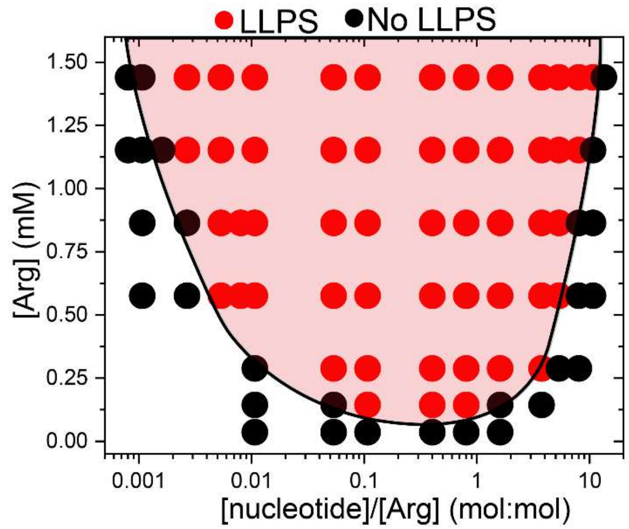

Figure S7. FUS^{RGG3}-poly(rU) isothermal state diagram. State diagram for FUS^{RGG3}-poly(rU) mixtures. This is identical data to Figure 1d, main-text, but plotted against arginine concentrations and nucleotide-to-arginine ratio. The sample buffer contains 25 mM Tris-HCl (pH 7.5), 150 mM NaCl and 20 mM DTT. The shaded red region represents the phase-separation regime and is drawn as a guide to the eye.

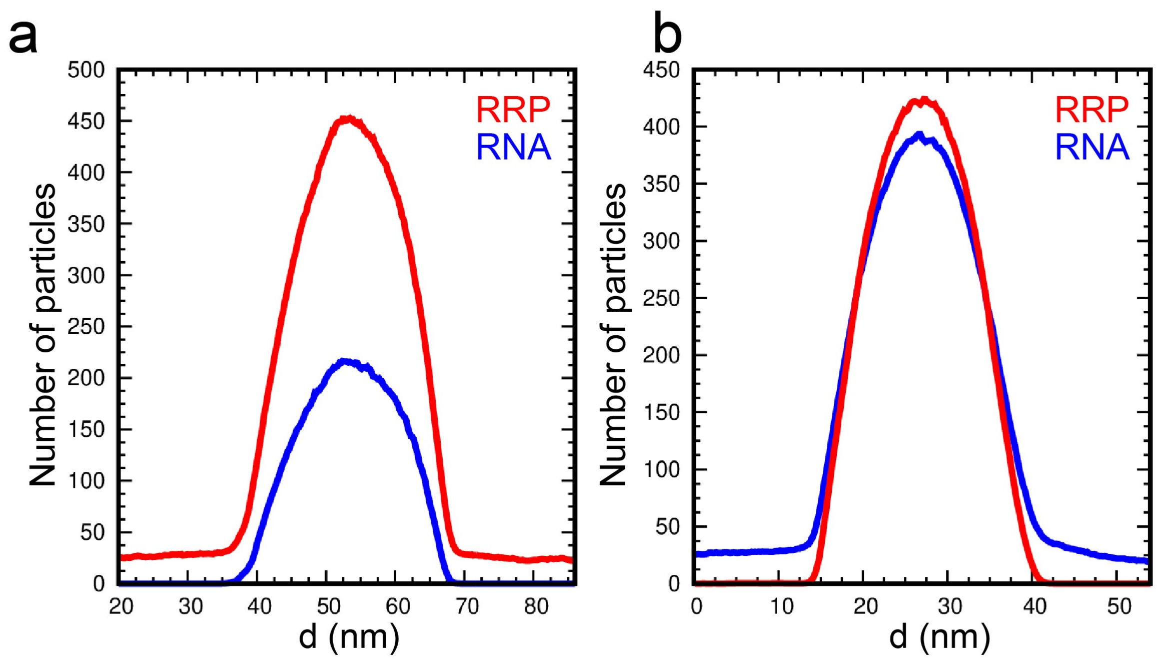

Figure S8. Density profiles of RNA and RRP from MD simulations. Density profiles for the RRP (FUS^{RGG3}) and poly(rU) RNA across RRP-RNA condensates from MD simulations at **(a)** $C_{RNA} < C_{RRP}$ **(b)** and $C_{RNA} > C_{RRP}$. These profiles correspond to the MD configurations shown in Figure 1d in the main-text. For both simulations, C_{RRP} = 1.3 mg/ml and the RNA-to-RRP (wt/wt) ratio is 0.5 for (a) and 1.7 for (b).

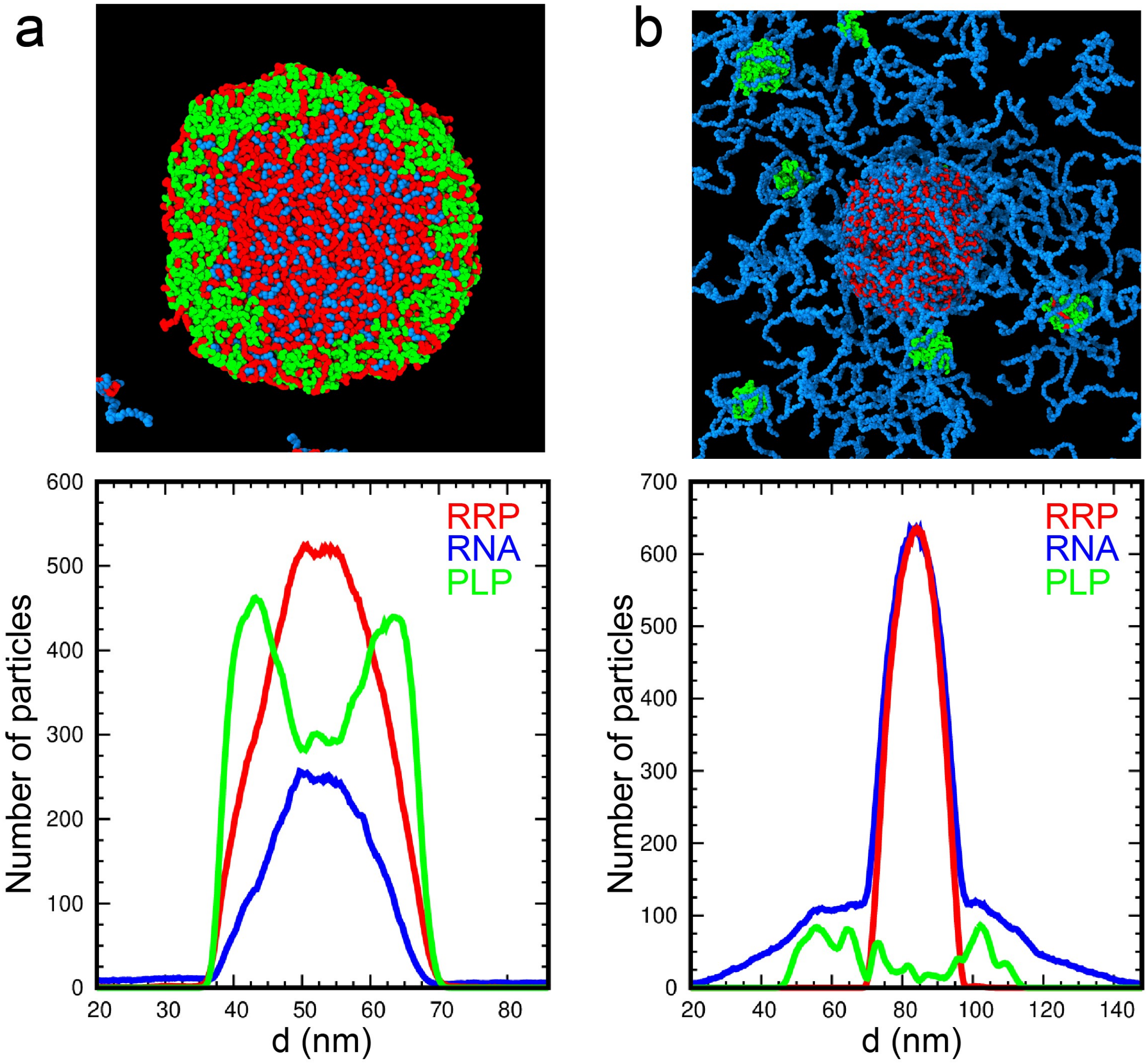

Figure S9. Density profiles of RNA, RRP, and PLP from MD simulations. Equilibrium configurations from MD simulations and the corresponding density profiles for the RRP (FUS^{RGG3}), PLP (FUS^{PLP}), and poly(rU) RNA across RRP-RNA condensates at **(a)** $C_{RNA} < C_{RRP}$ and **(b)** $C_{RNA} > C_{RRP}$. The recruitment of PLP is enhanced at $C_{RNA} < C_{RRP}$ with a visible localization of PLP chains on the surface of the RRP-RNA condensates. For both simulations, C_{RRP}= 1.3 mg/ml, C_{PLP}= 0.4 mg/ml and the RNA-to-RRP ratio (wt/wt) is 0.5 for (a) and 1.7 for (b).

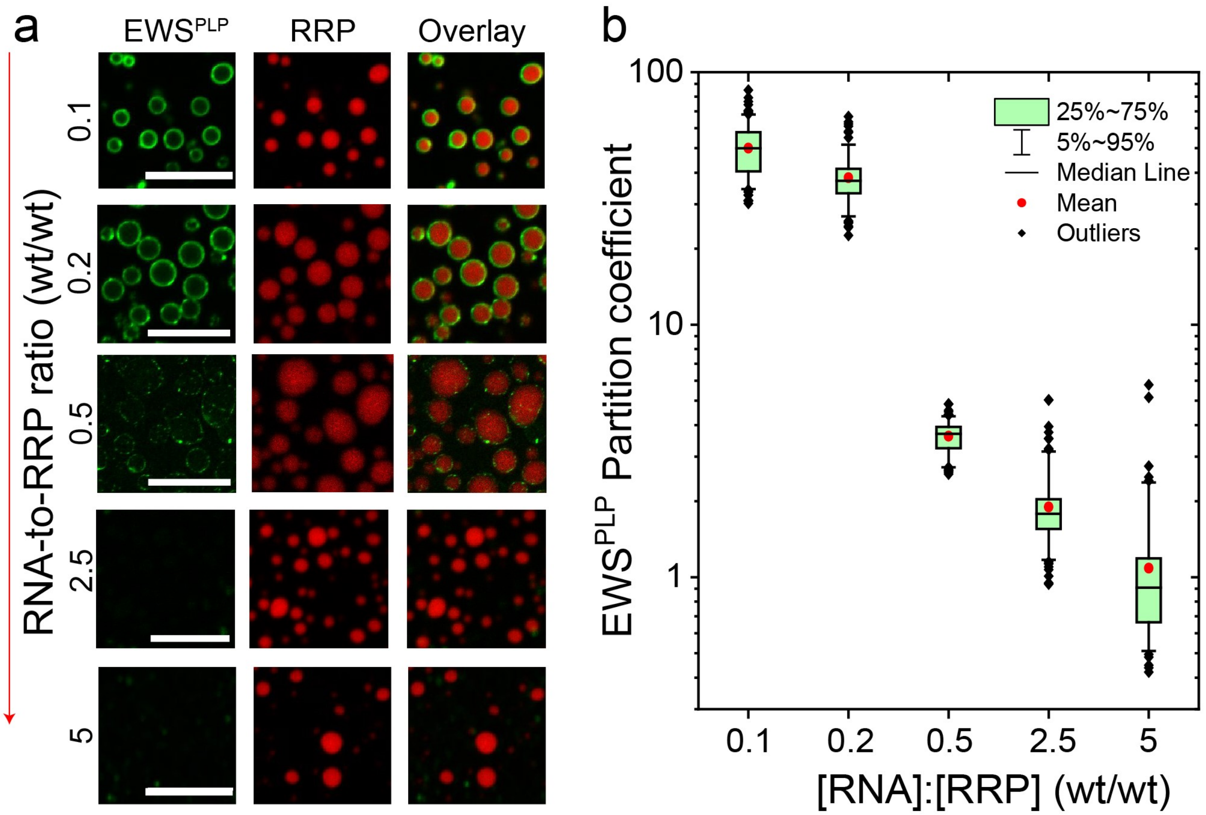

Figure S10. EWS^{PLP} preferentially partitions into the surface of RRP-rich RRP-RNA condensates. Multicolor confocal fluorescence microscopy images **(a)** and partition coefficients box plot **(b)** showing that EWS^{PLP} (labeled with Alexa488) is recruited into RNA-RRP [poly(rU)-FUS^{RGG3}] droplets at low RNA-to-RRP ratio while at high RNA-to-RRP ratio, PLP partitioning significantly decreases. poly(rU)-FUS^{RGG3} condensates were prepared at FUS^{RGG3}=1 mg/ml (with ~ 1% Alexa594-labeled peptide) and varying poly(rU)-to-FUS^{RGG3} ratio. The number of droplets (n) analyzed across different samples for partition is n = 100. Source data are provided as a Source Data file. Scale bars represent 10 μm. The sample buffer contains 25 mM Tris-HCl (pH 7.5), 150 mM NaCl and 20 mM DTT.

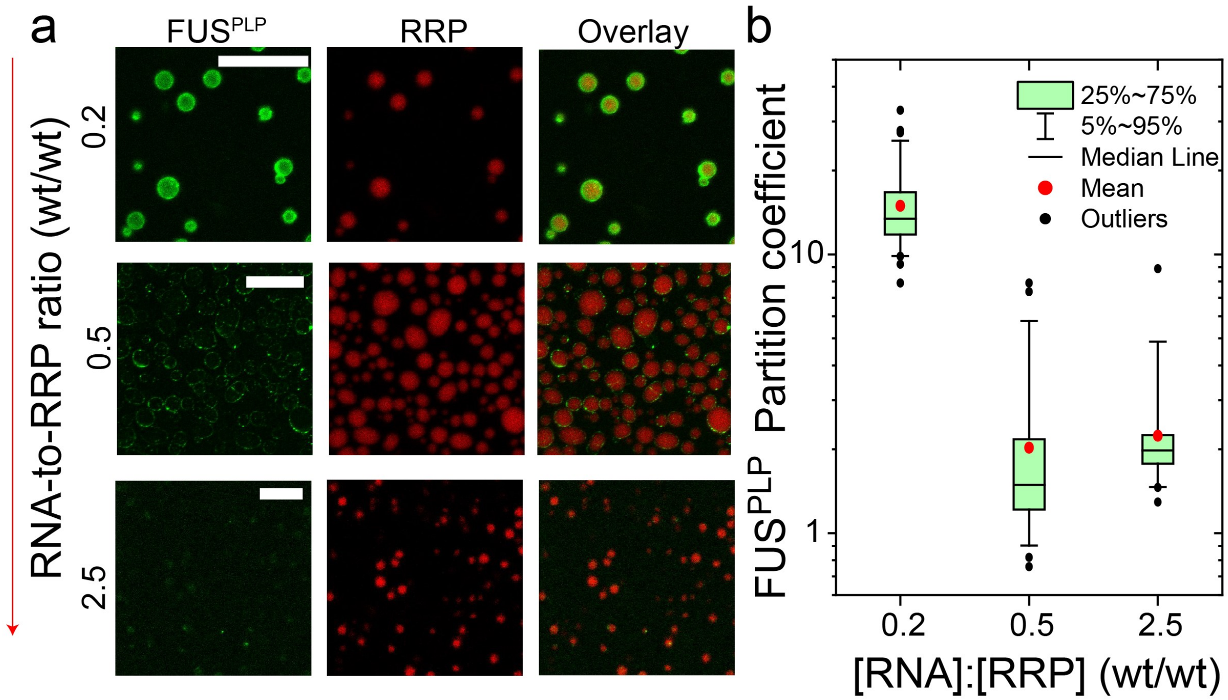

Figure S11. FUS^{PLP} shows preferential partitioning into RRP-rich RRP-RNA condensates. Multicolor confocal fluorescence microscopy images **(a)** and partition coefficient box plot **(b)** showing that FUS^{PLP} is recruited into RNA-RRP [poly(rU)-FUS^{RGG3}] droplets at low RNA-to-RRP ratio while at high RNA-to-RRP, PLP (labeled with Alexa488) partitioning significantly decreases. poly(rU)-FUS^{RGG3} condensates were prepared at FUS^{RGG3}= 1 mg/ml (with ~ 1% labeled:unlabeled ratio of Alexa594-FUS^{RGG3}) and varying poly(rU)-to-FUS^{RGG3} ratio. The number of droplets (n) analyzed across different samples for partition coefficient calculation is n = 50. Source data are provided as a Source Data file. Scale bars represent 10 μm. The sample buffer contains 25 mM Tris-HCl (pH 7.5), 150 mM NaCl and 20 mM DTT.

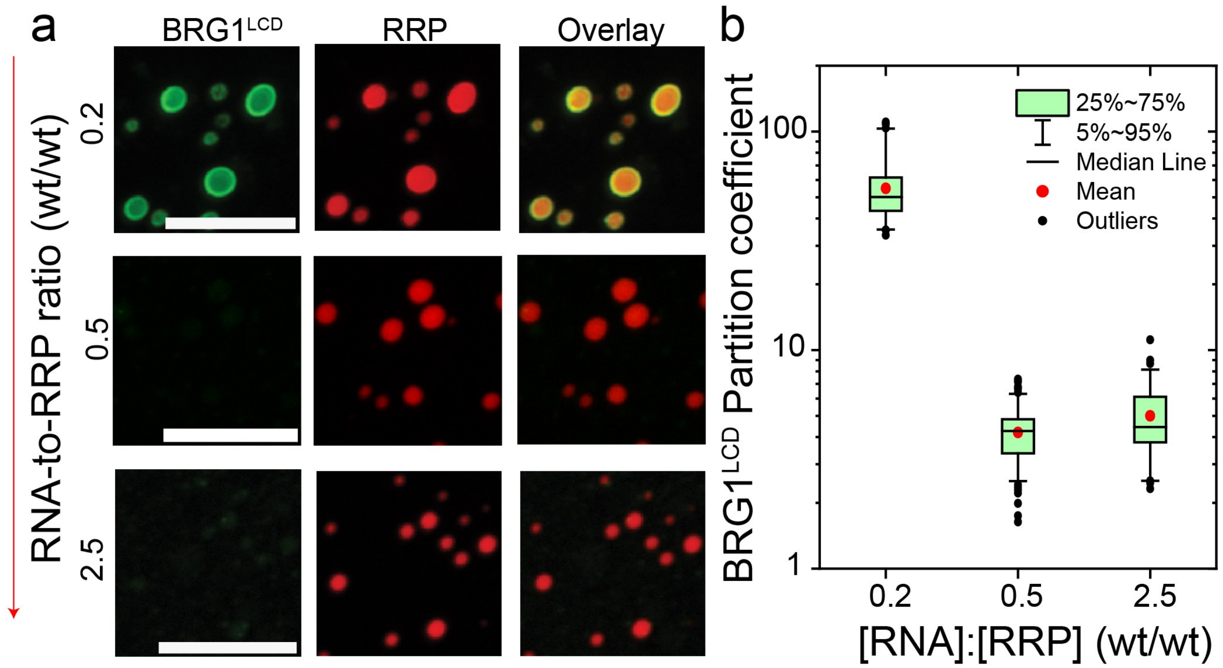

Figure S12. BRG1^{LCD} shows preferential partitioning into RRP-rich RRP-RNA condensates. Multicolor confocal fluorescence microscopy images **(a)** and partition coefficient box plot **(b)** showing that BRG1^{LCD} is recruited into RNA-RRP [poly(rU)-FUS^{RGG3}] droplets at low RNA-to-RRP ratio while at high RNA-to-RRP, BRG1^{LCD} (labeled with Alexa488) does not show any preferential partitioning. Poly(rU)-FUS^{RGG3} condensates were prepared at FUS^{RGG3}=1 mg/ml (with ~ 1% labeled:unlabeled Alexa594-FUS^{RGG3}) and varying poly(rU)-to-FUS^{RGG3} ratio. The number of droplets (n) analyzed across different samples for partition coefficient calculation is n = 75. Source data are provided as a Source Data file. Scale bars represent 10 μm. The sample buffer contains 25 mM Tris-HCl (pH 7.5), 150 mM NaCl and 20 mM DTT.

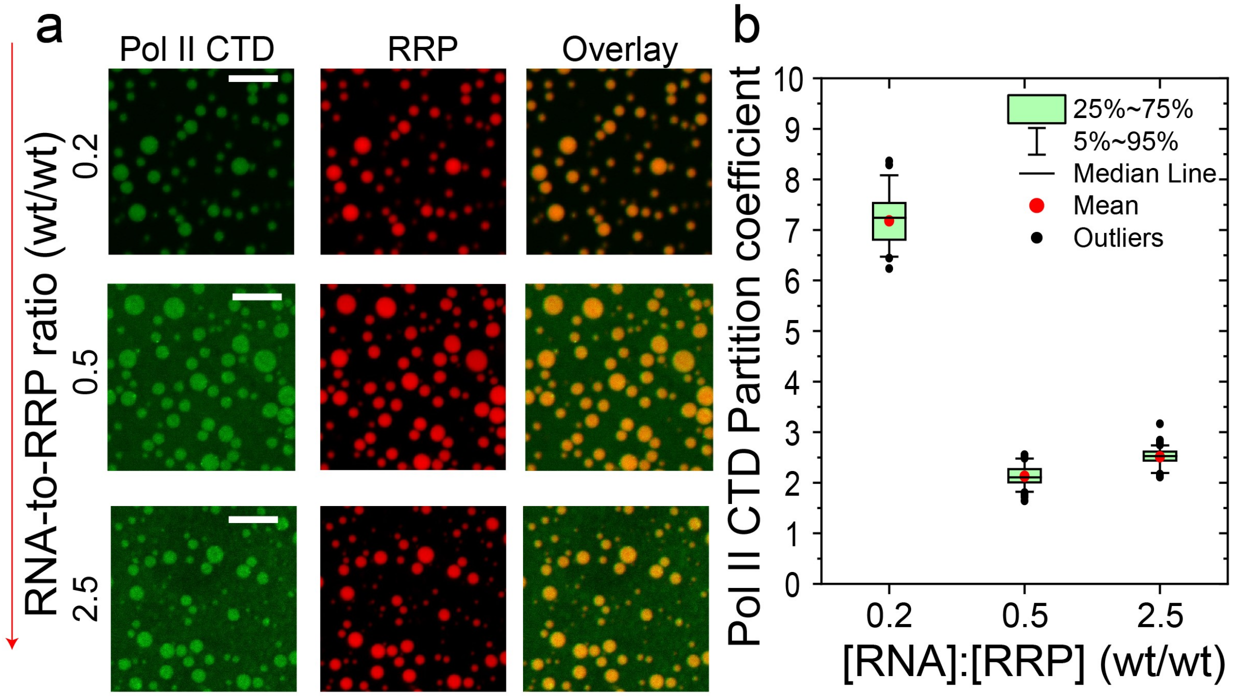

Figure S13. RNA Pol II CTD preferentially partitions into RRP-rich RRP-RNA condensates. Multicolor confocal fluorescence microscopy images **(a)** and partition coefficients box plot **(b)** showing that Pol II CTD (labeled with Alexa488) is recruited into RRP-RNA [poly(rU)-FUS^{RGG3}] droplets at low RNA-to-RRP ratio while at high RNA-to-RRP, Pol II CTD shows relatively lower partitioning. poly(rU)-FUS^{RGG3} condensates were prepared at FUS^{RGG3}= 1 mg/ml (with ~1% labeled:unlabeled Alexa594-FUS^{RGG3}) and varying poly(rU)-to-FUS^{RGG3} ratio, as indicated. The number of droplets (n) analyzed across different samples for partition coefficient calculation is n = 45. Source data are provided as a Source Data file. Scale bars represent 10 μm. The sample buffer contains 25 mM Tris-HCl (pH 7.5), 150 mM NaCl and 20 mM DTT.

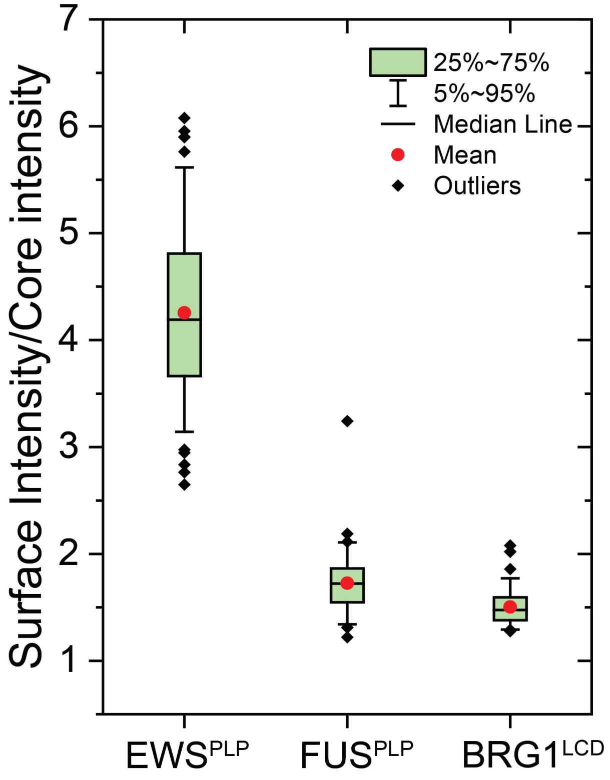

Figure S14. Surface versus core recruitment of PLP clients in RRP-RNA condensates. Box plot showing the ratio of surface and core intensity of PLP clients (EWS^{PLP}, FUS^{PLP}, and $BRG1^{LCD}$) within RRP-RNA [poly(rU)-FUS^{RGG3}] droplets at low RNA-to-RRP ratio. poly(rU)-FUS^{RGG3} condensates were prepared at FUS^{RGG3} = 1 mg/ml and poly(rU)-to-FUS^{RGG3} ratio of 0.2 (wt/wt).The number of droplets (n) analyzed across different samples is n = 70. The sample buffer contains 25 mM Tris-HCl (pH 7.5), 150 mM NaCl and 20 mM DTT. Source data are provided as a Source Data file.

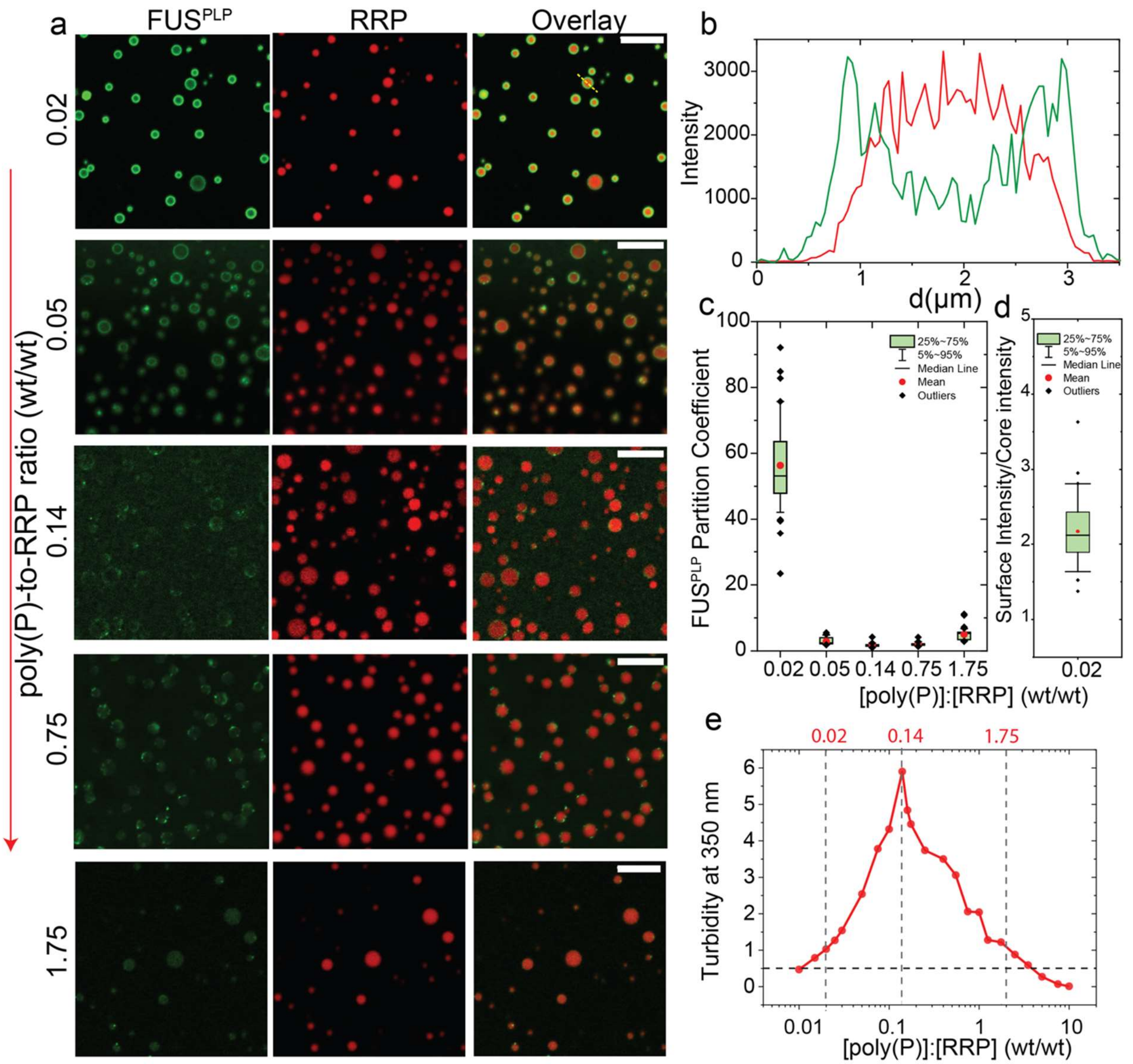

Figure S15. FUS^{PLP} shows preferential partitioning into RRP-rich RRP-poly(phosphate) condensates. Multicolor confocal fluorescence microscopy images **(a)**, intensity profile **(b),** and partition coefficient box plot **(c)** showing that FUS^{PLP} is recruited into poly(P)-RRP [poly(P)-FUS^{RGG3}] droplets at low poly(P)-to-RRP ratio while at high poly(P)-to-RRP, PLP (labeled with Alexa488) partitioning significantly decreases. poly(P)-FUS^{RGG3} condensates were prepared at FUS^{RGG3}= 1 mg/ml (with ~ 1% labeled:unlabeled ratio of Alexa594-FUS^{RGG3}) and varying poly(P)-to-FUS^{RGG3} ratio. **(d)** Box plot showing the ratio of surface and core intensity of FUS^{PLP} within RRP-poly(P) [poly(P)-FUS^{RGG3}] droplets at poly(P)-to-RRP ratio of 0.02 (wt/wt). **(e)** Turbidity at 350 nm for FUS^{RGG3}-poly(P) mixtures prepared at FUS^{RGG3} concentration of 1.0 mg/ml and variable poly(P) concentrations. The intensity profile in (b) is for poly(P)-to-RRP ratio of 0.02. The number of droplets (n) analyzed across different samples for box plots (c&d) is n = 50. Scale bars represent 10 μm. The sample buffer contains 25 mM Tris-HCl (pH 7.5), 150 mM NaCl and 20 mM DTT. Source data are provided as a Source Data file for c&d.

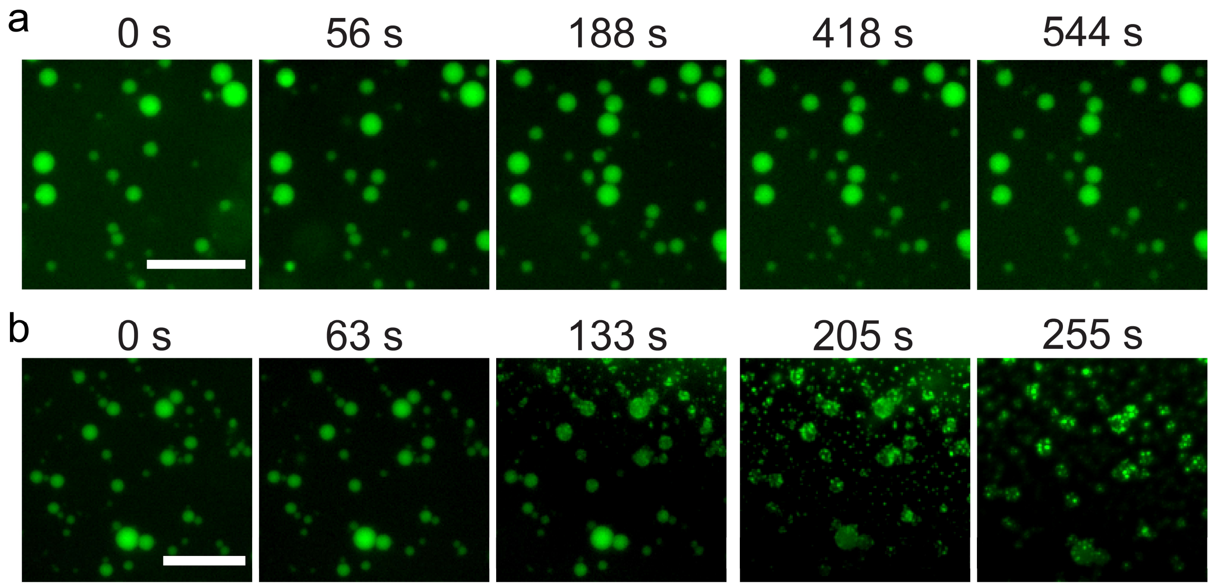

Figure S16. RNA mediated PLP-RRP demixing behavior is not impacted by sample volume change. (a) Time-lapse microscopy images after the addition of buffer to a sample containing PLP-RRP condensates showing that the PLP-RRP droplets are not affected by the volume change. The sample was prepared at [FUS^{PLP}] =250 μM and [FUS^{RGG3}]=750 μM (2.6 mg/ml). 1 μL of buffer was added to the 4 μL droplet sample. **(b)** Time-lapse microscopy images after the addition of poly(rU) RNA to a sample containing PLP-RRP condensates showing the sequestering of RRP (i.e. FUS^{RGG3}) from the PLP-RRP droplets. The sample was prepared at identical concentrations to those shown in (a). 0.3 μL of poly(rU) stock solution was added to the 4 μL droplet sample, resulting in a 6.5 mg/ml final concentration of poly(rU) RNA. ~ 500 nM Alexa488-labeled FUS^{RGG3} was used for imaging. Scale bar = 20 μm. The microscopy images (a&b) are representative of two independent sample replicates. The sample buffer contains 25 mM Tris-HCl (pH 7.5), 150 mM NaCl and 20 mM DTT.

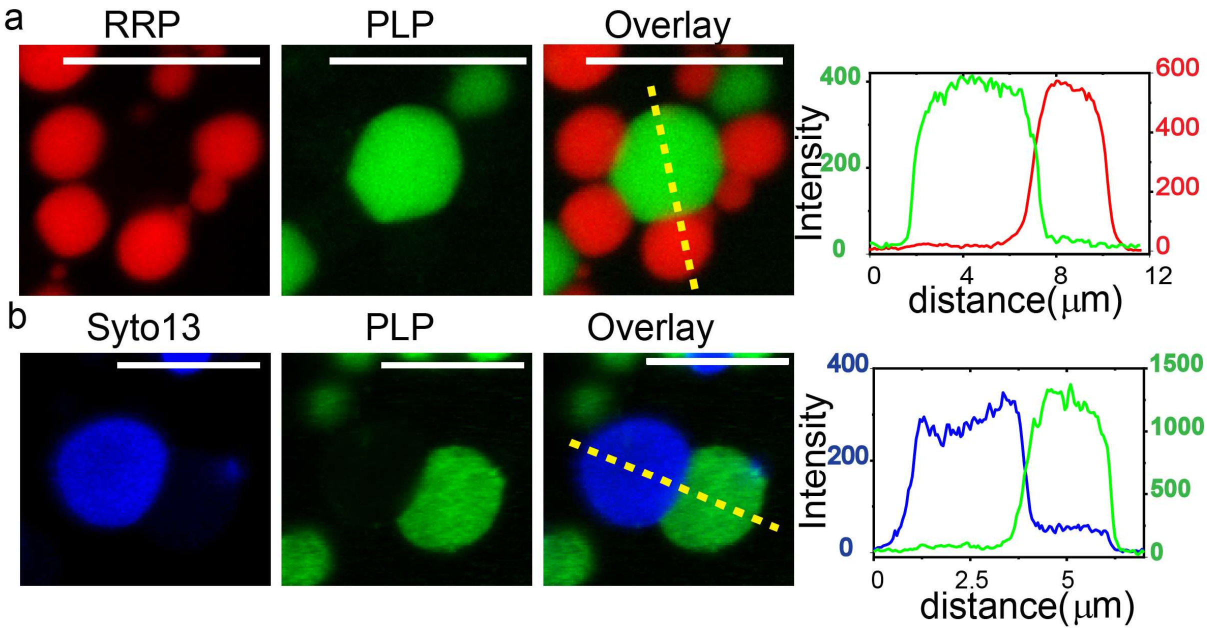

Figure S17. PLP condensates' coexistence with RRP-RNA condensates. (a) Multicolor confocal fluorescence microscopy images and intensity profiles for co-existing homotypic FUS^{PLP} droplets and heterotypic RRP-RNA droplets. PLP droplets (prepared at [FUS^{PLP}] = 400 μM) are mixed with RRP-RNA droplets (prepared at $[RGRGG]_5$ = 4 mg/ml and [poly(rU)] = 10 mg/ml). For imaging, ~ 500 nM Alexa594-labeled $[RGRGG]_5$ and ~500 nM Alexa488-labeled FUS^{PLP} were used. Scale bars represent 10 μm. **(b)** Identical sample with RNA stained by SYTO13 nucleic acid-staining dye (for imaging FUS^{PLP} here, Cy5-labeled protein was used). Scale bars represent 4 μm. The microscopy images are representative of two independent sample replicates. The sample buffer contains 25 mM Tris-HCl (pH 7.5), 150 mM NaCl and 20 mM DTT.

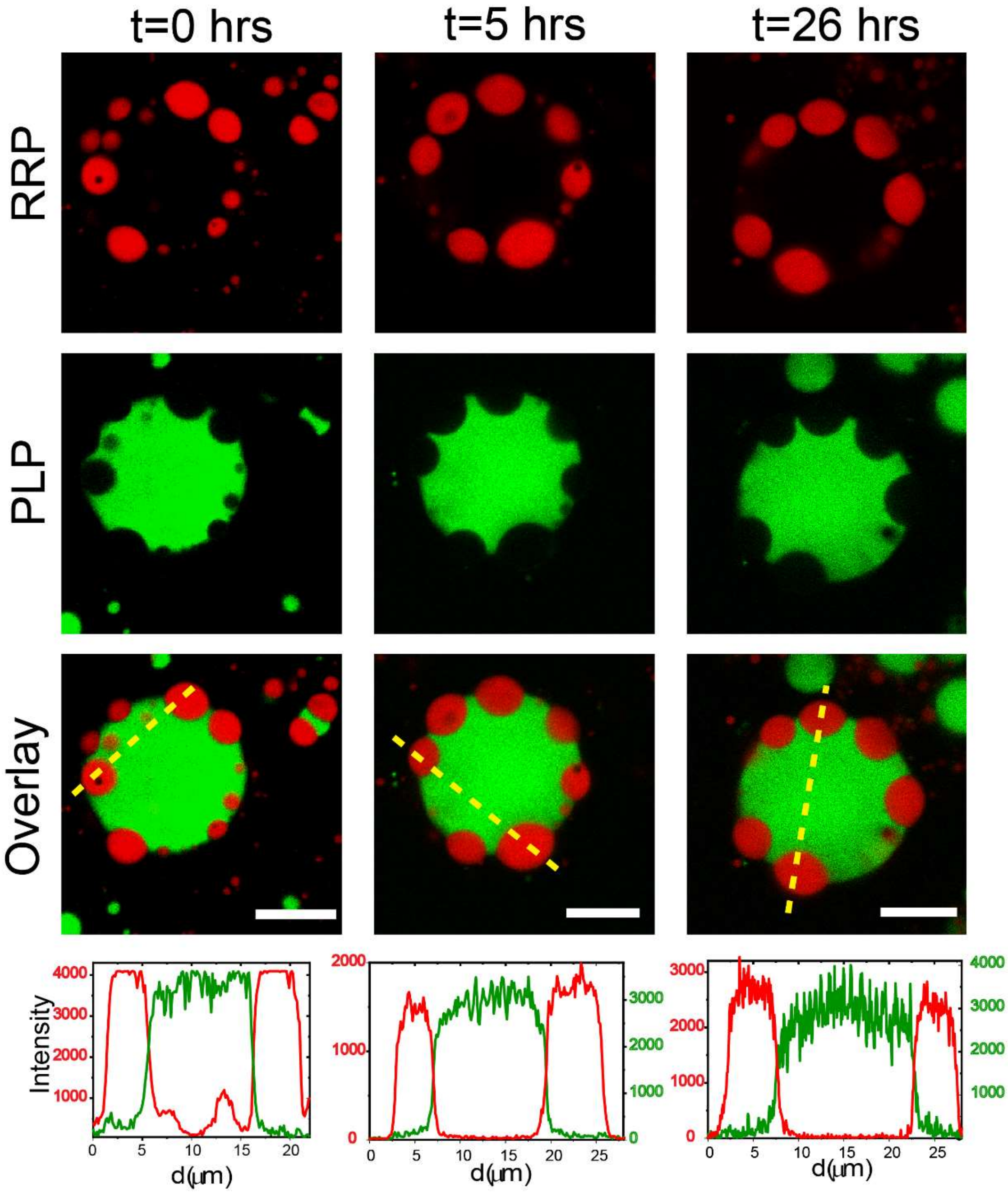

Figure S18. Stability of multi-phasic PLP-RRP-RNA condensates. Multicolor confocal fluorescence time-lapse images and intensity profiles for co-existing homotypic PLP droplets and heterotypic RRP-RNA droplets. The sample was prepared at [FUS^{PLP}]=400 μM, $[RGRGG]_5$=2 mg/ml and [poly(rU)]=5 mg/ml. For imaging, 500 nM Alexa594-labeled $[RGRGG]_5$ and 500 nM Alexa488-labeled FUS^{PLP} were used. Scale bar represents 10 μm. The reported images are representative of several replicates imaged from different spots in the same sample. The sample buffer contains 25 mM Tris-HCl (pH 7.5), 150 mM NaCl and 20 mM DTT.

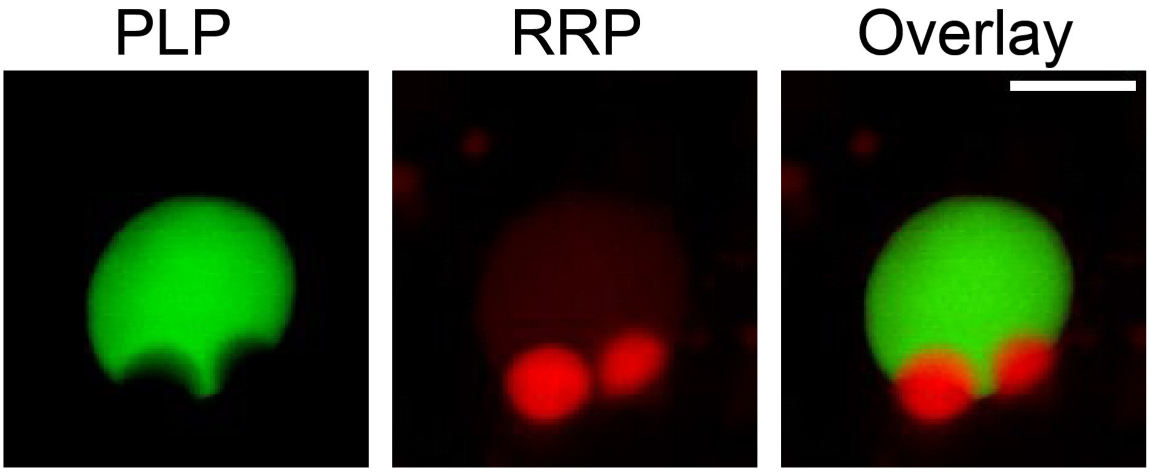

Figure S19. Multi-phasic PLP-RRP-RNA condensates with yeast total RNA. Multicolor confocal fluorescence images for co-existing homotypic PLP droplets and heterotypic RRP-RNA droplets using yeast total RNA (cellular RNA). The sample was prepared at [FUS^{PLP}] =400 μM, $[RGRGG]_5$=2 mg/ml and [yeast total RNA] = 1.5 mg/ml. For imaging, 500 nM Alexa594-labeled $[RGRGG]_5$ and 500 nM Alexa488-labeled FUS^{PLP} were used. Scale bar represents 5 μm. The reported images are representative of several replicates imaged from different spots in the same sample.The sample buffer contains 25 mM Tris-HCl (pH 7.5), 150 mM NaCl and 20 mM DTT.

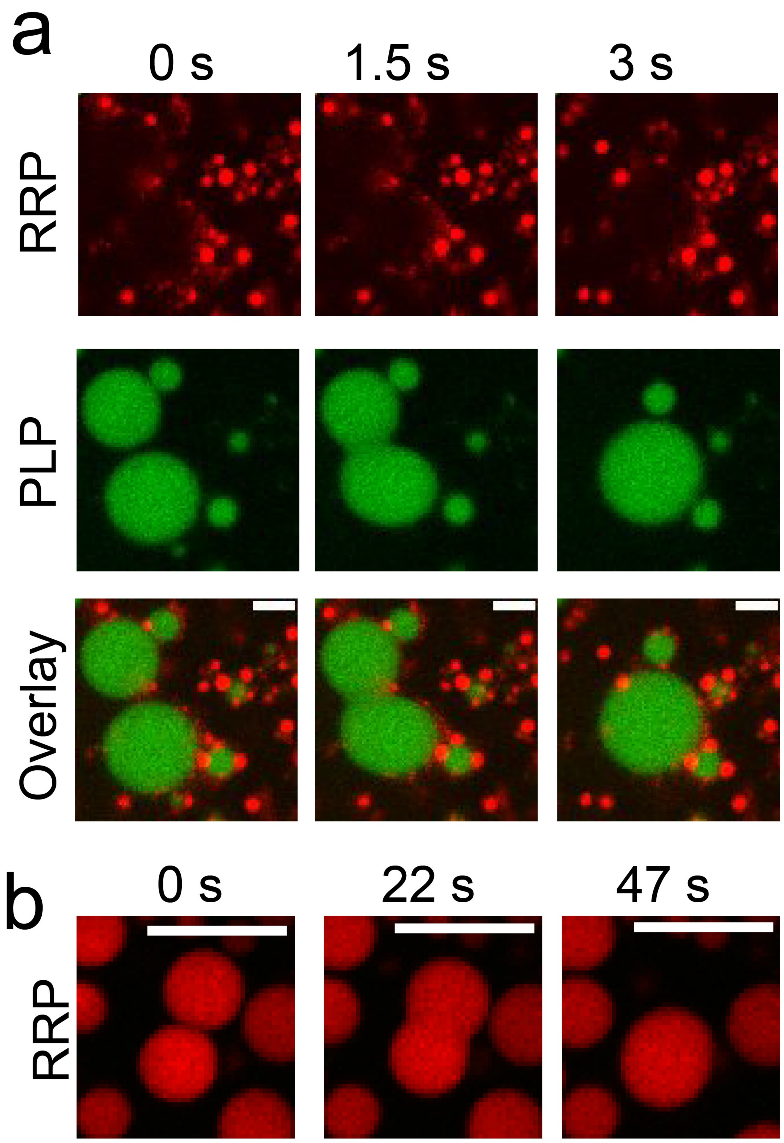

Figure S20. RRP-RNA condensates and PLP condensates show coalescence behavior. Multicolor confocal fluorescence time-lapse images showing the coalescence of **(a)** FUS^{PLP} condensates and **(b)** RRP (FUS^{RGG3})-RNA condensates in a PLP-RRP-RNA ternary mixture. For the data in **(a)**, the sample was prepared at [FUS^{PLP}]=250 μM, [FUS^{RGG3}]=750 μM (2.6 mg/ml) and [poly(rU)]=13.0 mg/ml. 500 nM Alexa488-labeled PLP and 500 nM Alexa594-labeled RRP were used for visualization. Scale bar represents 5 μm. For the data in **(b)**, The sample was prepared at [FUS^{PLP}] = 250 μM, [FUS^{RGG3}] = 750 μM (2.6 mg/ml) and [poly(rA)] = 6.5 mg/ml. Scale bar represents 8 μm. The microscopy images (a&b) are representative of two independent sample replicates. Both samples were prepared in a 25 mM Tris-HCl (pH 7.5), 150 mM NaCl and 20 mM DTT buffer.

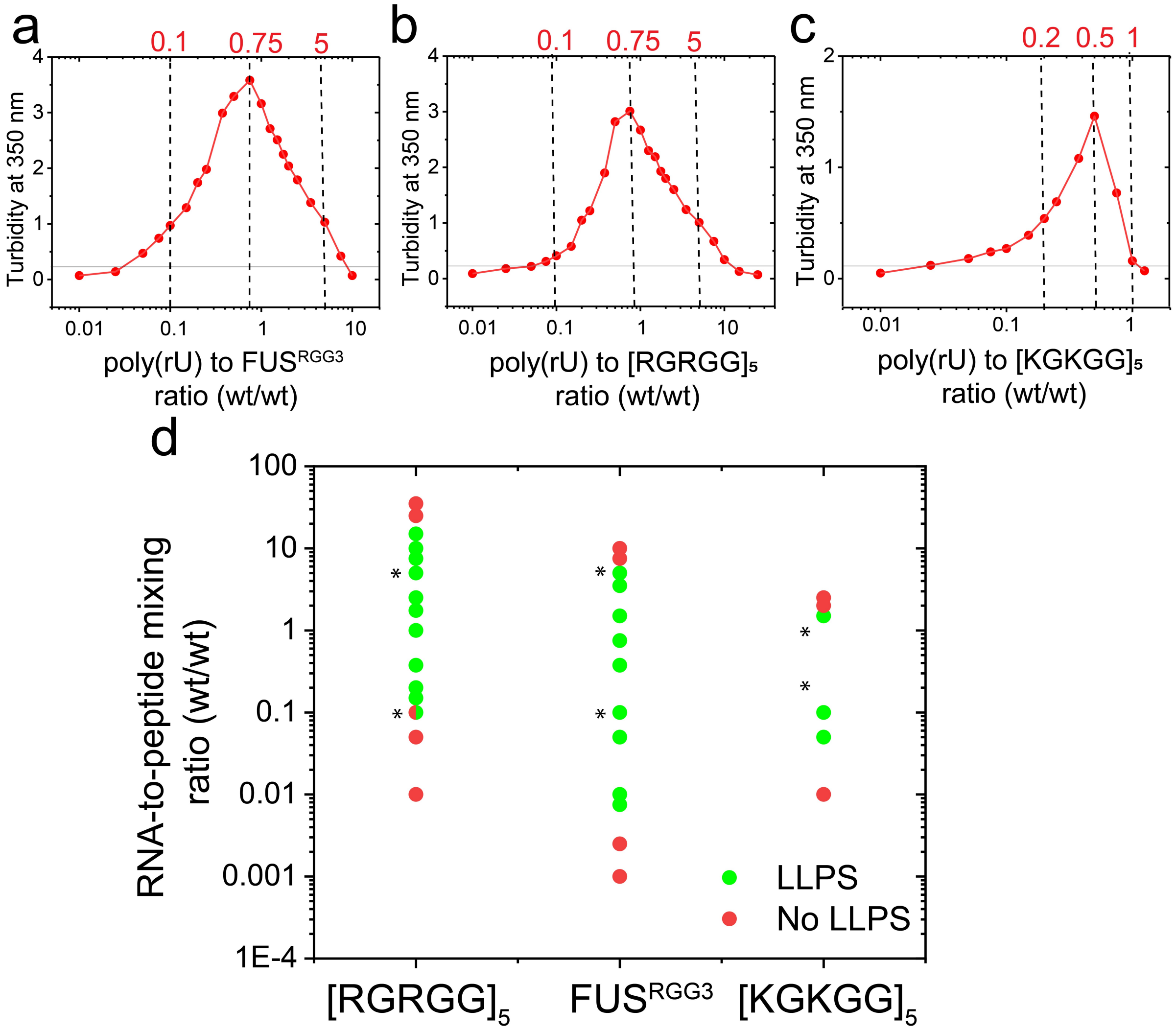

Figure S21. RRP and KRP undergo reentrant condensation with RNA. (a) Turbidity at 350 nm for FUS^{RGG3}-poly(rU) mixtures prepared at FUS^{RGG3} concentration of 0.347 mg/ml and variable poly(rU) RNA concentrations. **(b)** Replotting[2] of the turbidity at 350 nm for $[RGRGG]_5$-poly(rU) mixtures prepared at $[RGRGG]_5$ concentration of 0.24 mg/ml and variable poly(rU) RNA concentrations. **(c)** Replotting[2] of the turbidity at 350 nm for $[KGKGG]_5$-poly(rU) mixtures prepared at $[KGKGG]_5$ concentration of 0.22 mg/ml and variable poly(rU) RNA concentrations. **(d)** Phase strips[2] of peptide-RNA mixtures. For each strip, the peptide concentration was fixed at 0.24, 0.3, and 0.22 mg/ml for $[RGRGG]_5$, FUS^{RGG3}, and $[KGKGG]_5$, respectively. poly(rU) RNA concentration was varied. The sample phase separation state (LLPS or no LLPS) was determined via optical microscopy. The asterisks represent the minimum and maximum mixing ratios used for the various experiments. $[RGRGG]_5$ and $[KGKGG]_5$ samples were prepared in a buffer containing 25 mM Tris-HCl (pH 7.5) and 20 mM DTT. FUS^{RGG3} samples were prepared in a buffer containing 25 mM Tris-HCl (pH 7.5), 150 mM NaCl and 20 mM DTT.

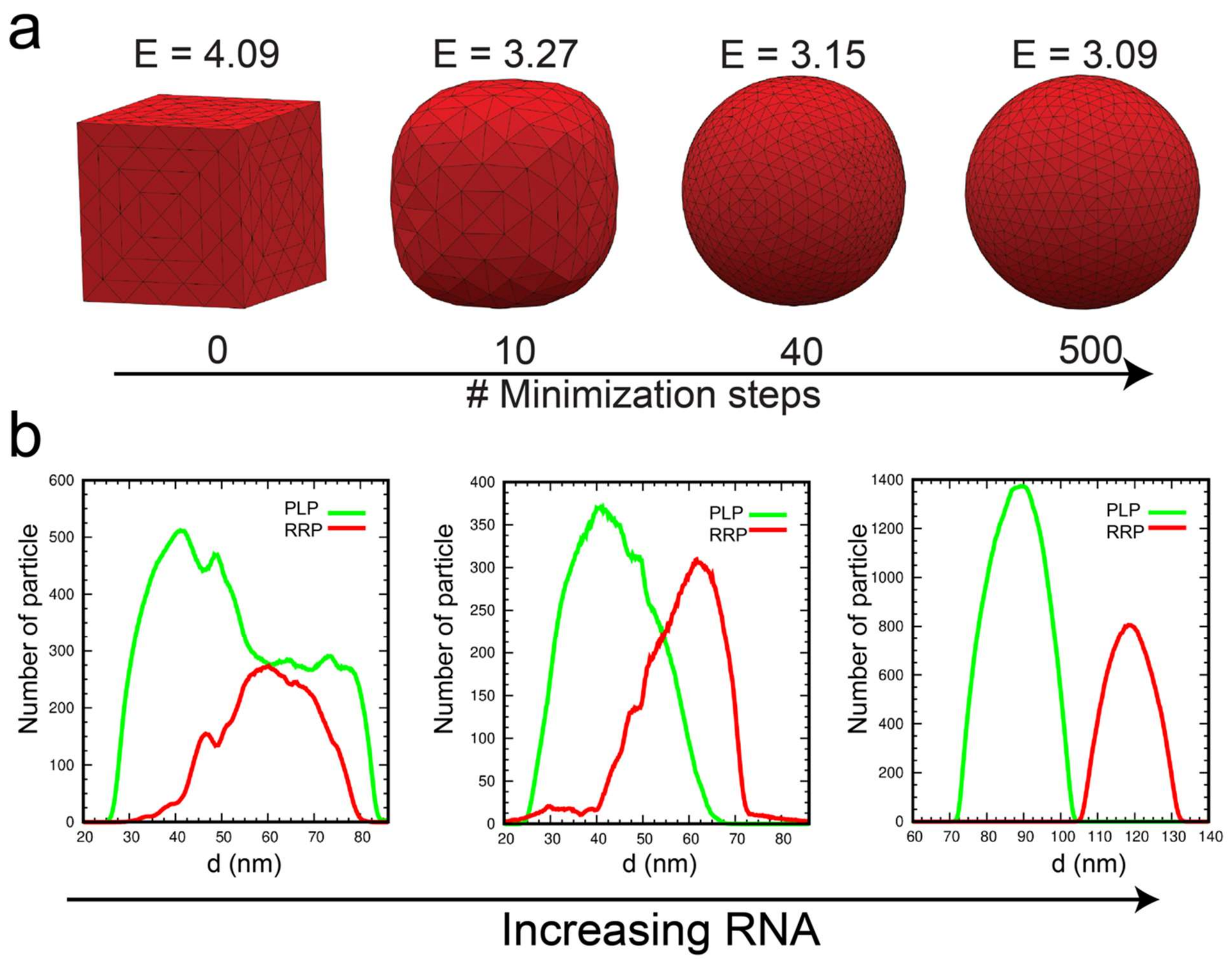

Figure S22. A simple illustration of the fluid-interface modeling simulation and density profiles for MD simulations. (a) Time evolution of a cube of liquid with 50 mN/m surface tension using Surface Evolver. The minimization of interfacial energy leads to the transition from a cube to a spherical droplet, which is the geometry with the least surface energy. **(b)** Density profiles for the molecular dynamics simulation snapshots are shown in Figure 4f in the main text. For simulation details, see the legend of Figure 4f in the main text.

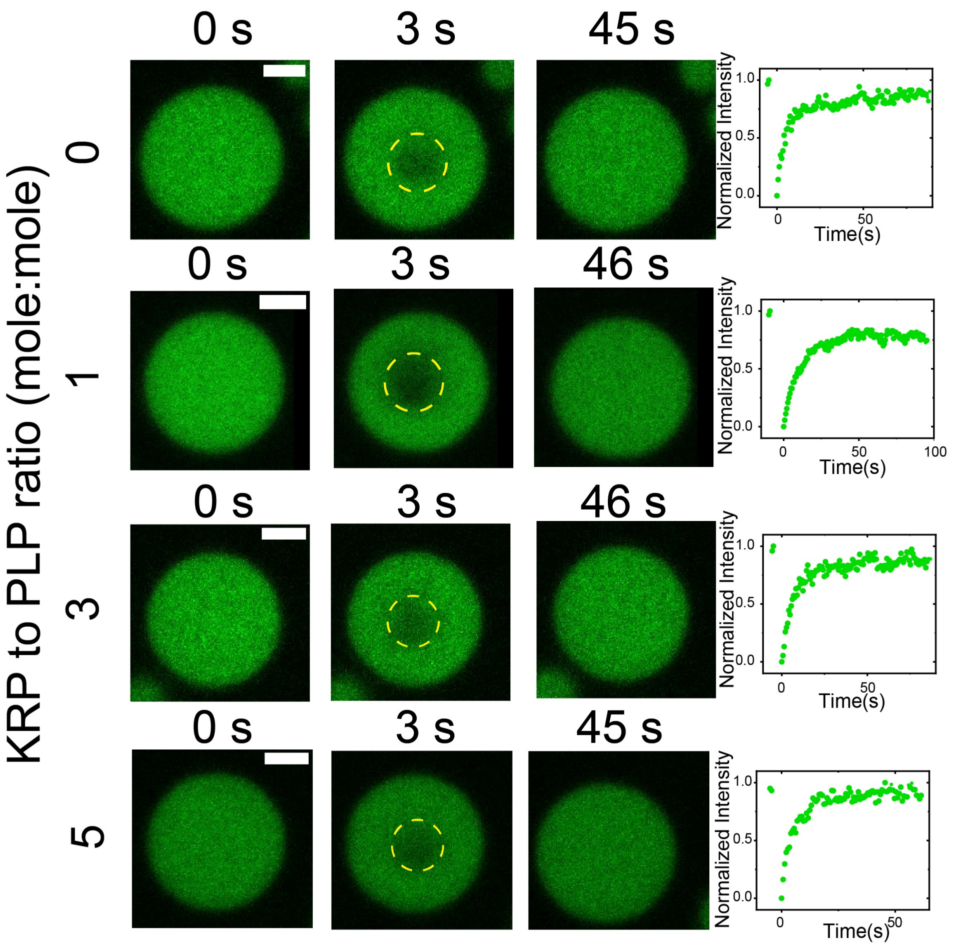

Figure S23. Representative FRAP images and intensity time traces for PLP-KRP condensates. Time-lapse FRAP images (left) and intensity time traces (right) for PLP-KRP condensates prepared at a fixed PLP concentration and variable KRP ($[KGKGG]_5$) to PLP ratio. For all samples, FUS^{PLP} concentration is fixed at 280 µM. The sample buffer contains 25 mM Tris-HCl (pH 7.5), 150 mM NaCl and 20 mM DTT. Scale bars represent 5µm. Bleaching occurs at t=3s. The microscopy images are representative of at least three FRAP events from different spots in the same sample. The imaging/FRAP assay was performed utilizing ~1% Alexa488-labeled PLP (labeled: unlabeled ratio).

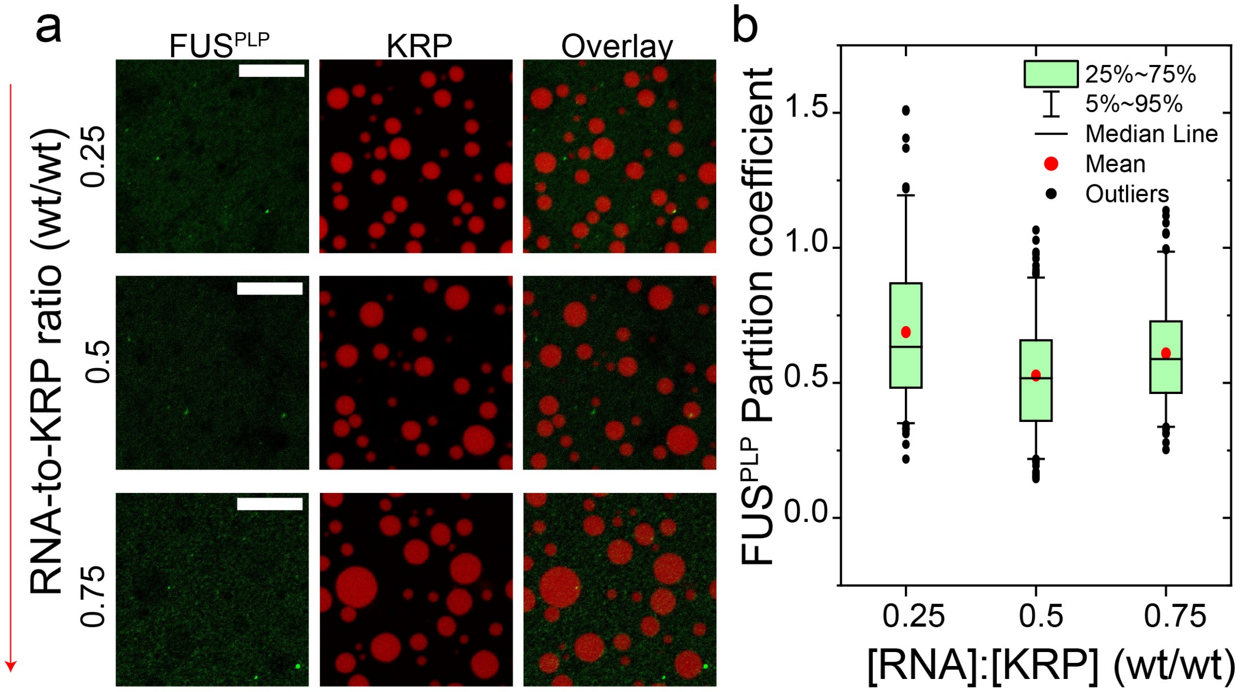

Figure S24. PLP does not partition into KRP-RNA condensates across all mixture compositions. Multicolor confocal fluorescence microscopy images (**a**) and partition coefficient box plot (**b**) of Alexa488-labeled FUS^{PLP} in KRP-RNA droplets. KRP-RNA condensates were prepared at a fixed KRP ($[KGKGG]_5$) concentration of 1 mg/ml and variable RNA [poly(rU)] to KRP ratio, as indicated. Scale bars represent 10 µm. The number of droplets (n) analyzed across different samples for partition is n = 150. Source data are provided as a Source Data file. The sample buffer contains 25 mM Tris-HCl (pH 7.5), 150 mM NaCl and 20 mM DTT.

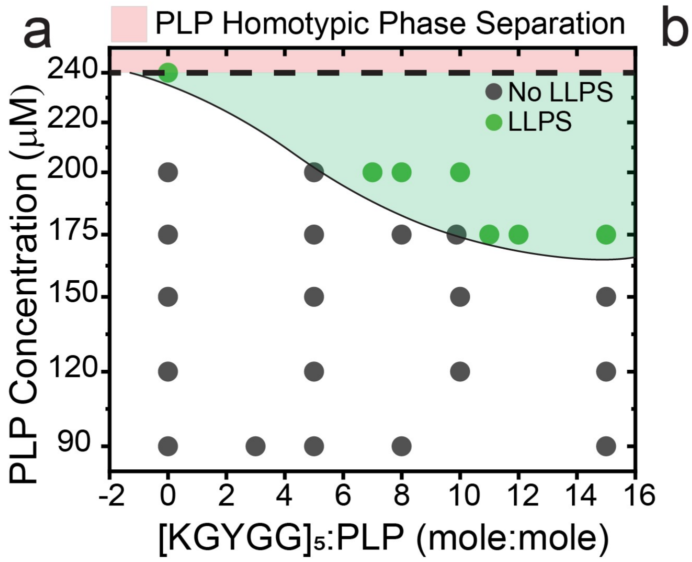

Figure S25. A tyrosine-variant of KRP restores PLP binding ability and stabilizes a shared fluid-fluid interface. (a) Isothermal state diagram of FUS^{PLP}-$[KGYGG]_5$ mixtures showing that $[KGYGG]_5$ facilitates PLP phase-separation. The shaded green region shows the heterotypic phase separation regime for PLP-$[KGYGG]_5$ mixtures while the shaded pink region denotes the PLP homotypic phase separation regime (saturation concentration ~240 μM). Both shaded regions are drawn as a guide to the eye. All samples were prepared in a 25 mM Tris-HCl, 150 mM NaCl and 20 mM DTT buffer. **(b)** Multicolor confocal fluorescence microscopy images and intensity profile for co-existing homotypic FUS^{PLP} droplets and heterotypic $[KGYGG]_5$-RNA droplets. Each type of droplet was separately prepared at initial concentrations of $[FUS^{PLP}]$=400 μM , $[KGYGG]_5$=4 mg/ml and [poly(rU)]=0.4 mg/ml and then mixed (1:1 vol/vol). The reported images are representative of several replicates imaged from different spots in the same sample. For imaging, 1% Alexa594-labeled $[KGYGG]_5$ and Alexa488-labeled FUS^{PLP} were used (labeled: unlabeled ratio). Scale bar represents 5 μm.

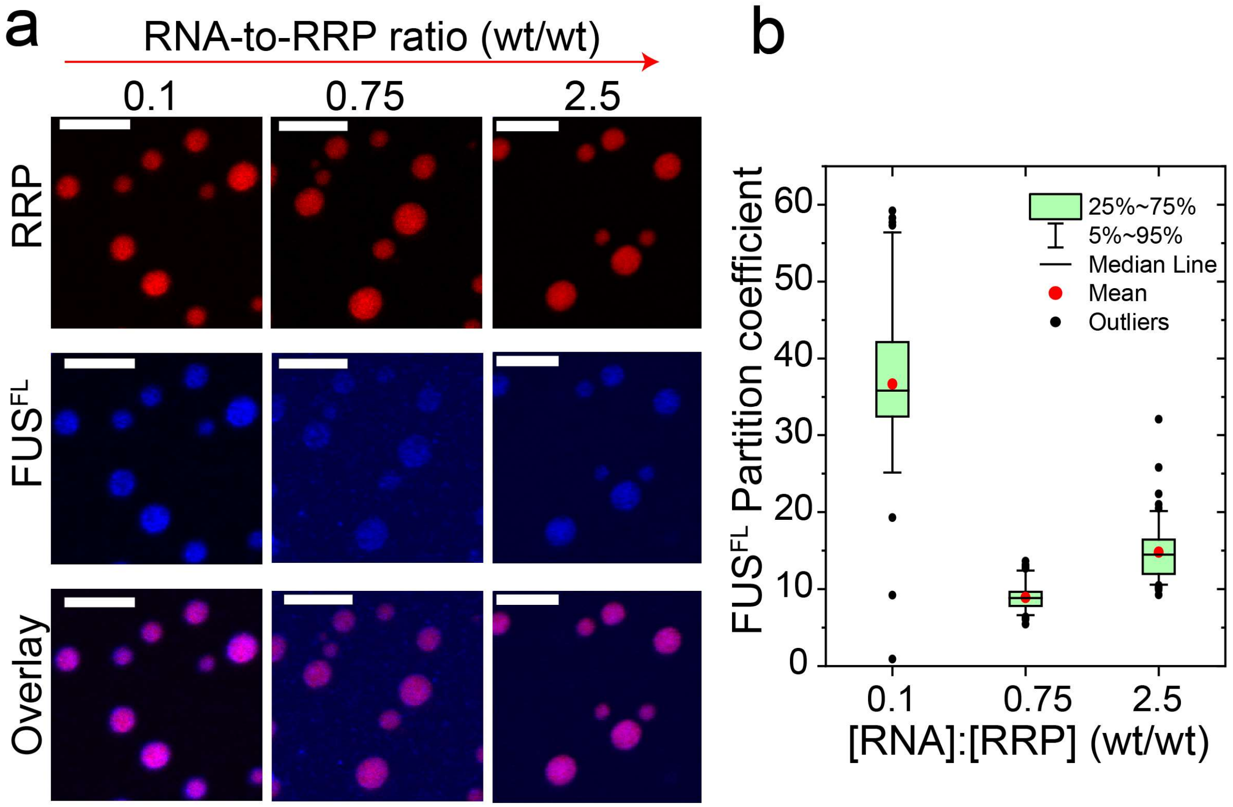

Figure S26. Full-length FUS (FUS^{FL}) partitions into RRP-RNA condensates across all mixture compositions. Multicolor confocal fluorescence microscopy images **(a)** and partition coefficient box plot **(b)** showing the partition of FUS^{FL} (labeled with Alexa488) in RNA-RRP droplets at varying RNA-to-RRP ratio. RNA-RRP condensates were prepared at FUS^{RGG3}=1 mg/ml (labeled with Alexa594) and varying RNA [poly(rU)] to FUS^{RGG3} ratio. The number of droplets (n) analyzed across different samples for partition is n = 80. Source data are provided as a Source Data file. Scale bars represent 5 μm. The samples were prepared in a buffer containing 25 mM Tris-HCl (pH 7.5), 150 mM NaCl, and 20 mM DTT. Compare this data with Figure 1h&k in the main-text.

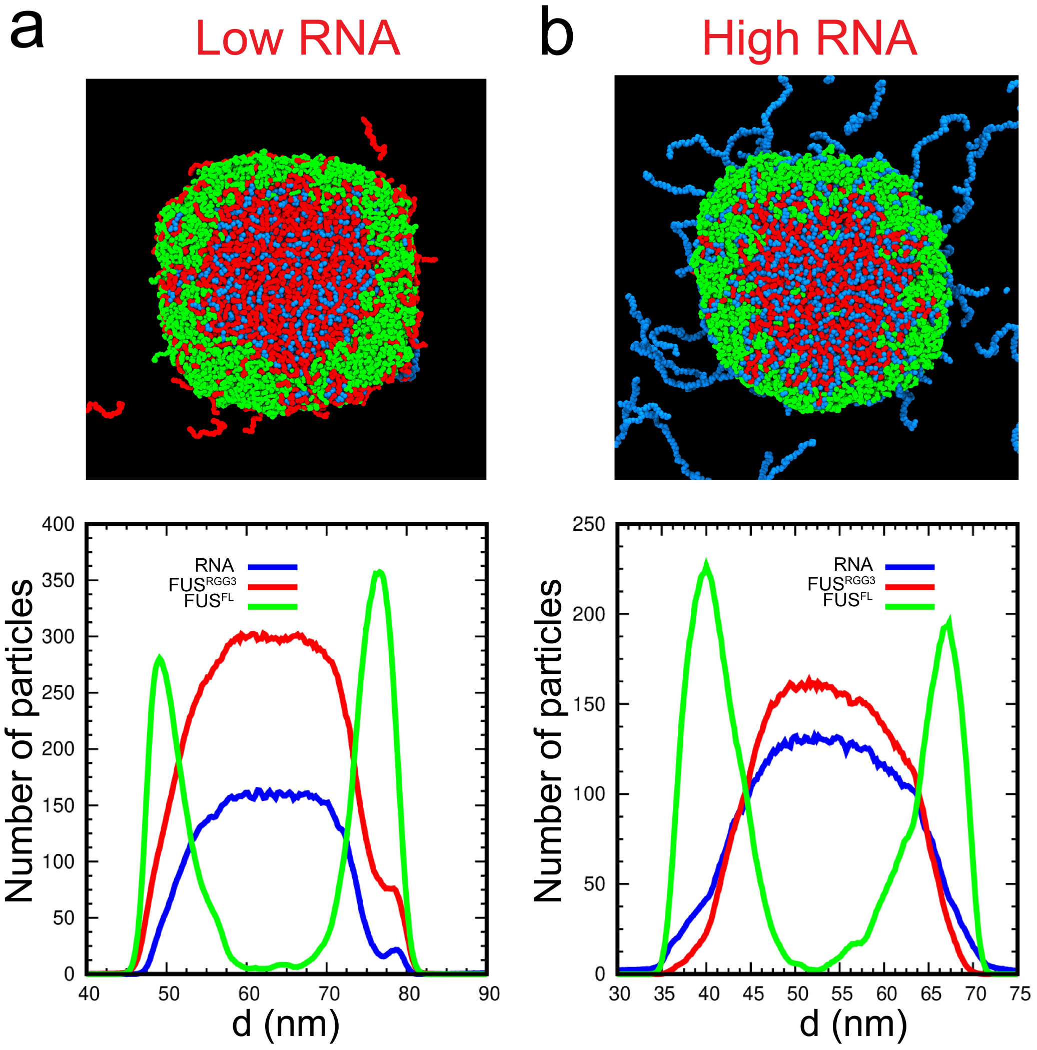

Figure S27. Molecular dynamics simulation snapshots showing the partition of FUS^{FL} in RRP-RNA droplets. Representative equilibrium configurations and corresponding density profiles obtained from molecular dynamics simulation of RRP-RNA condensates at both low **(a)** and high **(b)** RNA-to-RRP mixing ratios. FUS^{FL} accumulates at the surface of RRP-RNA condensates in both conditions due to its ability to interact with RRP chains (through PLP-RRP interactions) and RNA chains (through RBD-RNA interactions, see Fig. 5e, main-text). RNA is visualized as blue chains, RRP is visualized as red chains, and FUS^{FL} is visualized as green chains. For both simulations, C_{RRP}=1.3 mg/ml, C_{FUS}=0.7 mg/ml and RNA-to-RRP ratio (wt/wt) of 0.5 (left) and 2.0 (right).

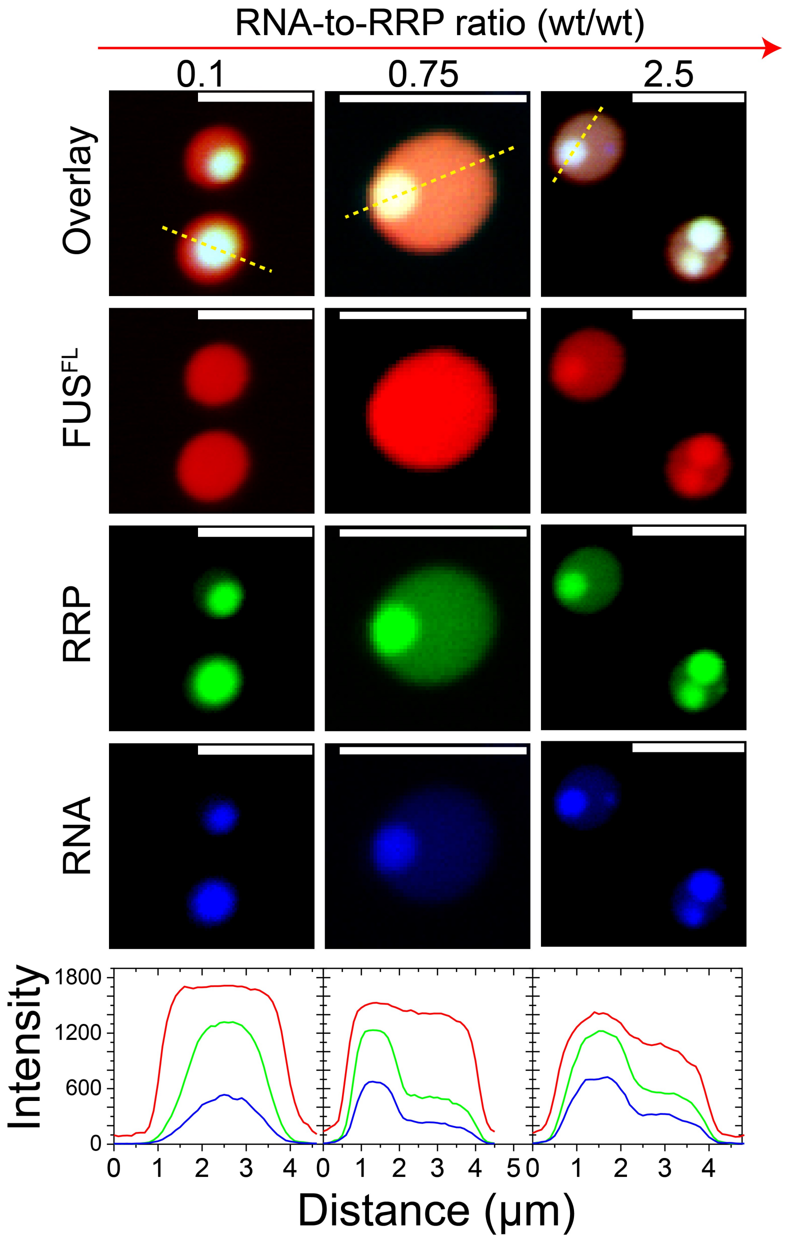

Figure S28. FUS^{FL} condensates completely engulf RRP-RNA condensates across all mixture compositions. Multicolor confocal fluorescence microscopy images and intensity profiles for co-existing homotypic FUS^{FL} droplets (red; Cy5-labeled FUS^{PLP}) and heterotypic RRP (green; Alexa594-labeled FUS^{RGG3}) and poly(rU) RNA (blue; probed by SYTO13) condensates at different RNA-to-RRP ratio. Each type of droplet was separately prepared at [FUS^{FL}]= 21.3 μM, [FUS^{RGG3}] = 1 mg/ml and varying RNA-to-RRP ratios, as indicated, and then mixed (1:1 vol/vol). All samples were made in a buffer containing 25 mM Tris-HCl (pH 7.5), 150 mM NaCl, and 20 mM DTT. The microscopy images are representative of two independent sample replicates. All fluorescent probes were added at a 1% labeled: unlabeled ratio. All scale bars represent 5 μm.

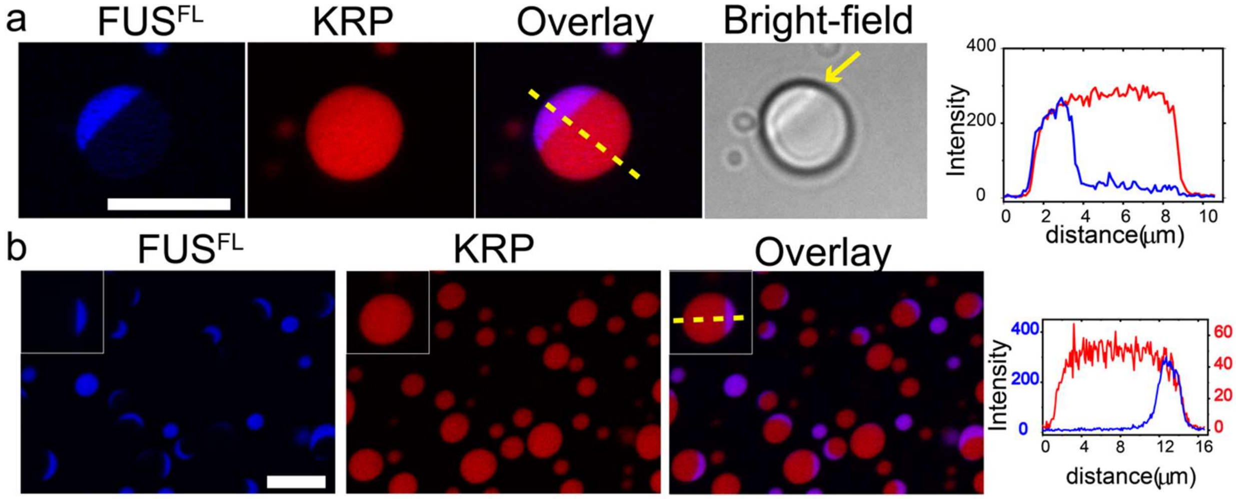

Figure S29. The Janus-like architecture of FUS-KRP-RNA condensates. Multicolor confocal fluorescence and bright-field microscopy images and intensity profiles for Janus droplets formed by homotypic FUS^{FL} droplets (blue) and heterotypic KRP-RNA condensates (red). Each type of droplet was separately prepared at initial concentrations of $[FUS^{FL}]$=22 µM , $[KGKGG]_5$=4 mg/ml and RNA [poly(rU)]=3 mg/ml keeping $[KGKGG]_5$ to poly(rU) ratio at 0.75 (wt/wt) and then mixed (1:1 vol/vol). For imaging, 500 nM Alexa594-labeled $[KGKGG]_5$, 500 nM Alexa488-labeled FUS^{FL} were used for **(a)**; and 500 nM Alexa594-labeled $[KGKGG]_5$, 500 nM Alexa488-labeled FUS^{PLP} were used for **(b)**. The arrow in (a) points to the line separating the two lobes of the Janus droplet (visible in the bright-field channel). All samples were prepared in a buffer containing 25 mM Tris-HCl (pH 7.5), 150 mM NaCl, and 20 mM DTT. The reported images (a&b) are representative of several replicates imaged from different spots in the same sample. Scale bar represents 10 µm. Inset shows the zoomed-in appearance of a Janus droplet.

References

1 Gasteiger, E. et al. in The proteomics protocols handbook 571-607 (Humana press, Hatfield, 2005).

2 Alshareedah, I. et al. Interplay between Short-Range Attraction and Long-Range Repulsion Controls Reentrant Liquid Condensation of Ribonucleoprotein–RNA Complexes. Journal of the American Chemical Society 141, 14593-14602, doi:10.1021/jacs.9b03689 (2019).
