# Supplementary material for: Sequence-encoded and composition-dependent protein-RNA interactions control multiphasic condensate morphologies
Source: Nat Commun. 2021 Feb 8;12:872. doi: 10.1038/s41467-021-21089-4 (PMC7870978; doi:10.1038/s41467-021-21089-4)
Supplement: Supplementary file 5 — Reporting Summary [file 41467_2021_21089_MOESM5_ESM.pdf]

nature research

Corresponding author(s): Priya R. Banerjee

Last updated by author(s): 01/06/2021

Reporting Summary

Nature Research wishes to improve the reproducibility of the work that we publish. This form provides structure for consistency and transparency in reporting. For further information on Nature Research policies, see our Editorial Policies and the Editorial Policy Checklist.

Statistics

For all statistical analyses, confirm that the following items are present in the figure legend, table legend, main text, or Methods section.

n/a	Confirmed	
☐	☒	The exact sample size (n) for each experimental group/condition, given as a discrete number and unit of measurement
☐	☒	A statement on whether measurements were taken from distinct samples or whether the same sample was measured repeatedly
☐	☒	The statistical test(s) used AND whether they are one- or two-sided<br>*Only common tests should be described solely by name; describe more complex techniques in the Methods section.*
☒	☐	A description of all covariates tested
☒	☐	A description of any assumptions or corrections, such as tests of normality and adjustment for multiple comparisons
☐	☒	A full description of the statistical parameters including central tendency (e.g. means) or other basic estimates (e.g. regression coefficient) AND variation (e.g. standard deviation) or associated estimates of uncertainty (e.g. confidence intervals)
☐	☒	For null hypothesis testing, the test statistic (e.g. F, t, r) with confidence intervals, effect sizes, degrees of freedom and P value noted<br>*Give P values as exact values whenever suitable.*
☒	☐	For Bayesian analysis, information on the choice of priors and Markov chain Monte Carlo settings
☒	☐	For hierarchical and complex designs, identification of the appropriate level for tests and full reporting of outcomes
☒	☐	Estimates of effect sizes (e.g. Cohen's d, Pearson's r), indicating how they were calculated

Our web collection on statistics for biologists contains articles on many of the points above.

Software and code

Policy information about availability of computer code

Data collection	Fluorescence imaging were performed either using a Zeiss Primovert inverted iLED microscope, or a Lumicks C-Trap microscope, or a Zeiss LSM710 laser scanning confocal microscope. FRAP experiments were performed using Zeiss LSM710 laser scanning confocal microscope. FCS experiments were performed using Lumicks C-Trap microscope.
Data analysis	ZEN (blue, v2.3) was used for image recording/processing using Zeiss Primovert microscope. Bluelake (v1.6.11) was used for image recording and processing using Lumicks C-Trap microscope. Data analyses were performed using Excel 2016, MATLAB (R2018a). Image analysis was performed using Fiji-ImageJ (version 1.52p). Data plots and statistical analysis were performed using OriginPro (2018b). Images were prepared using Adobe Illustrator CC 2019 (v23.0). Fluid-interface modeling were performed using Surface Evolver (v2.70). MD simulation was performed using HOOMD-blue (2.7.0). MD visualization was done using VMD (v1.9.4) and OVITO (v3.2.0). Custom Matlab scripts for FRAP analysis are available from authors upon reasonable request.

For manuscripts utilizing custom algorithms or software that are central to the research but not yet described in published literature, software must be made available to editors and reviewers. We strongly encourage code deposition in a community repository (e.g. GitHub). See the Nature Research guidelines for submitting code & software for further information.

Data

Policy information about availability of data

All manuscripts must include a data availability statement. This statement should provide the following information, where applicable:

- Accession codes, unique identifiers, or web links for publicly available datasets
- A list of figures that have associated raw data
- A description of any restrictions on data availability

All data supporting the findings of this study are included in this paper and the supplementary information. Source data are provided with this paper. Additional data are available from the corresponding author upon reasonable request.

Field-specific reporting

Please select the one below that is the best fit for your research. If you are not sure, read the appropriate sections before making your selection.

☒ Life sciences ☐ Behavioural & social sciences ☐ Ecological, evolutionary & environmental sciences

For a reference copy of the document with all sections, see nature.com/documents/nr-reporting-summary-flat.pdf

Life sciences study design

All studies must disclose on these points even when the disclosure is negative.

Sample size	The proteins, synthetic peptides and RNA are reconstituted and stored as concentrated stock solutions and then diluted to final concentrations. The sample size is 5-10 micro-liters for all the experiments. This sample size is sufficient for microscopy analysis especially that our samples are sandwiched into sealed small imaging chambers. For statistics, the sample size was chosen in accordance with the experimental feasibility and the resulting error-to-value ratio. For Partition, we chose 30 or more droplets per sample which results in good error-to-value ratio (<40%). For FRAP, we performed 2-6 FRAPS per sample. FRAP is not a stochastic measurement and hence large statistics are not needed for the purposes of our study. Contact angle measurements were done on at least 25 droplet pairs. In each measurement, we were able to replicate the results at least two times.
Data exclusions	No Data were excluded
Replication	All the results reported in the manuscript were successfully reproduced. Each experiment was done twice and resulted in the same findings. For FRAP measurements, the experiment was repeated at different spots in the same sample. For Partition measurements, the experiment was repeated at different spots in the same sample. For qualitative microscopy imaging, the results are replicated at least two times per sample for two independent sample preparation. For state diagrams, the transition points between LLPS and mixed regimes have been successfully replicated twice. Multiple protein batches were used to reproduce the findings of this work.
Randomization	Randomization is not relevant to this study because we are studying individual systems and not groups of systems or a specific population.
Blinding	Blinding is not relevant to this study because the experimental outcome does not depend on any decision making, therefore there is no inherent bias and blinding is not required.

Reporting for specific materials, systems and methods

We require information from authors about some types of materials, experimental systems and methods used in many studies. Here, indicate whether each material, system or method listed is relevant to your study. If you are not sure if a list item applies to your research, read the appropriate section before selecting a response.

Materials & experimental systems

n/a	Involved in the study	
☒	☐	Antibodies
☒	☐	Eukaryotic cell lines
☒	☐	Palaeontology and archaeology
☒	☐	Animals and other organisms
☒	☐	Human research participants
☒	☐	Clinical data
☒	☐	Dual use research of concern

Methods

n/a	Involved in the study	
☒	☐	ChIP-seq
☒	☐	Flow cytometry
☒	☐	MRI-based neuroimaging
