# Supplementary material for: Sequence-encoded and composition-dependent protein-RNA interactions control multiphasic condensate morphologies
Source: Nat Commun. 2021 Feb 8;12:872. doi: 10.1038/s41467-021-21089-4 (PMC7870978; doi:10.1038/s41467-021-21089-4)
Supplement: Supplementary file 6 — Description of additional supplementary files [file 41467_2021_21089_MOESM6_ESM.docx]

**Description of additional supplementary information**

**Title: Supplementary Movie S1.**

Description: Multicolor fluorescence microscopy video showing that the addition of poly(rU) RNA induces condensate switching effect wherein PLP-RRP condensates transition to RRP-RNA condensates. (PLP: green, RRP: red). The scale bar is 20 µm.

**Title: Supplementary Movie S2.**

Description: Multicolor fluorescence microscopy video showing that the addition of poly(rU) RNA causes the demixing of PLP and RRP from well-mixed PLP-RRP condensates to coexisting PLP homotypic condensates and RRP-RNA heterotypic condensates. (PLP: green, RRP: red). The scale bar is 10 µm.
